# Serologic Surveillance of Highly Pathogenic Avian Influenza Virus Subtype H5 in Wildlife, Northeast Germany, 2023–2025

**DOI:** 10.3201/eid3205.251555

**Published:** 2026-05

**Authors:** Anne Günther, Josefine Wassermann, Jonas Heck, Marin Bussi, Andrea Aebischer, Christoph Staubach, Hannes Bergmann, Fabian H. Leendertz, Martin Beer, Gereon Schares, Kerstin Wernike

**Affiliations:** Friedrich-Loeffler-Institut, Greifswald–Insel Riems, Germany (A. Günther, J. Wassermann, J. Heck, A. Aebischer, C. Staubach, H. Bergmann, M. Beer, G. Schares, K. Wernike); Utrecht University, Utrecht, the Netherlands (M. Bussi); Helmholtz Institute for One Health, Greifswald, Germany (F.H. Leendertz); University of Greifswald, Greifswald (F.H. Leendertz)

**Keywords:** highly pathogenic avian influenza, mammalian spillover, viruses, influenza, serology, zoonoses, respiratory infections, HPAIV H5, Germany

## Abstract

We tested wild ruminants, boar, and carnivores in northeast Germany for highly pathogenic avian influenza subtype H5 antibodies. Wild ruminants were seronegative, but 3.5% of boar and 12.5%–21.9% of carnivores were seropositive, indicating frequent spillover. Because such events might accelerate mammalian (and ultimately human) adaptation, sustained monitoring remains essential.

The shift of seasonal epizootics toward a perpetual enzootic in Europe became a milestone in the epidemiology of highly pathogenic avian influenza viruses (HPAIV) (*1*). Rooted in a common ancestral virus of the goose/Guangdong influenza lineage in Southeast Asia, the evolving diversity of subtype H5 genotypes in Europe paved the way for the ongoing panzootic, globally threatening domestic and wild birds alike and resulting in numerous mammalian spillover infections worldwide. A broad and increasing spectrum of terrestrial, semiaquatic, and marine or aquatic mammalian species is affected (*2*). Despite the recent developments of mastitis in domestic cattle through ascending udder infections with HPAIV H5 (*3*), the more constant interface over the years was direct, often alimentary, exposure to HPAIV H5–positive prey or food (*4*).

Infections in predatory or scavenging species frequently caused neurologic signs, including severe encephalitis as cause of death (*5*). The concern about increasing chances for spillover events from birds to mammals proved to be justified already in the early stage of enzootic HPAIV H5 in Europe. However, those studies also suggested a certain level of asymptomatic infections and the possibility of surviving exposure (*6*).

We compared HPAIV H5 antibody prevalences between mammal groups in Germany that had frequent contact opportunities (carnivores) and conceivable or unlikely contact opportunities (omnivores and herbivores). We identify frequently affected host groups and factors that ultimately favor the risk for infection in carnivores.

## The Study

During December 2023–February 2025, we collected samples from 644 hunted predator game (group I) and 343 hoofed game animals (groups II and III) in the context of an ongoing disease surveillance project in the federal state of Mecklenburg–Western Pomerania in northeast Germany. In particular, the offshore islands and the mainland coast form important avian migratory areas but also breeding habitats with a high overall species diversity and abundance of wild birds. Many of those species have been affected during various HPAI H5 epizootics and the recent enzootic (*7*).

Predator game consisted of 5 species of the taxonomic families Canidae, Procyonidae, and Mustelidae (Table). Cervidae, Bovidae, and Suidae form groups II and III with five different species in total (1 sampled individual animal remains unspecified). We screened nasal swab and, for group I, lung and brain samples (n = 980) by using quantitative reverse transcription PCR for influenza A virus (IAV) RNA (Appendix Table 1). All 980 samples tested negative.

**Table:** Overview of species sampled for serologic and molecular screening for highly pathogenic avian influenza subtype H5 antibodies, grouped according to feeding behavior, northeast Germany, December 2023–February 2025*

Group and species	Serologic screening						Molecular screening	
	Total no. animals tested	No. IAV antibody– positive	IAV antibody–positive, % (95% CI)	No. H5 antibody–positive	H5 antibody–positive, % (95% CI)		Total no. animals tested†	No. IAV RNA–positive
Carnivores (group I)	606‡	120	19.8 (16.7–23.2)	100§	16.5 (13.6–19.7)		644	0
(Red) fox (*Vulpes vulpes*)	354	73	20.6 (16.5–25.2)	58	16.4 (12.7–20.7)		376	0
Raccoon dog (*Nyctereutes procyonoides*)	179	29	16.2 (11.1–22.4)	27	15.1 (10.2–21.2)		194	0
Raccoon (*Procyon lotor*)	64	17	26.6 (16.3–39.1)	14	21.9 (12.5–34.0)		65	0
European badger (*Meles meles*)	8	1	12.5 (0.3–52.7)	1	12.5 (0.3–52.7)		8	0
European pine marten (*Martes martes*)	1	0	0 (0.0–97.5)	NT	NT		1	0
Omnivores, wild boar (*Sus scrofa*) (group II)	115	6	5.2 (1.9–11.0)	4	3.5 (0.9–NT8.7)		114	0
Herbivores (group III)	228	0	0 (0.0–1.6)	NT	NT		222	0
European fallow deer (*Dama dama*)	106	0	0 (0.0–3.4)	NT	NT		106	0
Roe deer (*Capreolus capreolus*)	91	0	0 (0.0–4.0)	NT	NT		91	0
Red deer (*Cervus elaphus*)	23	0	0 (0.0–14.8)	NT	NT		23	0
Mouflon (*Ovis gmelini musimon*)	7	0	0 (0.0–40.9)	NT	NT		1	0
Unknown	1	0	0 (0.0–97.5)	NT	NT		1	0

*Results refer to screenings on generic antibodies against IAV or specific for subtype H5 on the basis of 2 subsequent multispecies ELISA tests. Quantitative reverse transcription real-time PCR results shown for swab or organ tissue samples tested for influenza A virus RNA. IAV, influenza A virus; NT, not tested. †Includes 1 omnivore and 6 herbivores with blood sample only and no swab or organ tissue sample for molecular analyses. ‡Includes 38 animals with swab or organ tissue sample only and no blood sample for serologic analyses. §Includes 6 IAV antibody–positive samples not tested for H5 antibody because of limited sample volume.

Nucleoprotein (NP)–based ELISA indicated no seropositivity against IAV in wild ruminants, whereas 5.2% (95% CI 1.9%–11.0%) of the wild boar (*Sus scrofa*) and all carnivorous species, except the single tested European pine marten (*Martes martes*), tested positive (Table) (Appendix). The subsequent ELISA for detection of H5-specific antibodies revealed 3.5% (95% CI 0.9%–8.7%) seropositive wild boar and 12.5% (95% CI 0.3%–52.7%) to 21.9% (95% CI 12.5%–34.0%) seropositive carnivorous species (Table; Appendix Table 1), thereby confirming most of the seropositivity caused by an IAV of subtype H5. The percentage of non–H5 IAV antibody-positive animals ranged from 4.7% (95% CI 0.9%–13.1%) in raccoons (*Procyon lotor*) and 4% (95% CI 2.4%–6.9%) in (red) foxes (*Vulpes vulpes*) to 1.7% (95% CI 0.2%–6.1%) in wild boar and 1.1% (95% CI 0.1%–3.9%) in raccoon dogs (*Nyctereutes procyonoides*). We also tested a subset of NP-antibody positive or negative samples through serum neutralization test to confirm the presence or absence of antibodies against subtype H5 (Appendix). We confirmed the ELISA findings in a representative subset of 8 IAV-positive but H5-negative carnivore samples and compared them with 4 H5-positive samples, whereas H5 seropositivity in wild boar samples remained below the detection limit of the serum neutralization test.

Wild ruminants represent mammalian species with rather unlikely contact possibilities to potentially infected wild birds and, as herbivores, no apparent interface in their diet. However, direct exposure cannot be ruled out, and low susceptibility might be another plausible explanation for our consistent seronegative findings. At the other end of the food chain, our serologic results suggest previous exposure of carnivores to subtype H5 viruses. Raccoons, foxes, and raccoon dogs represent the species with the highest proportion of seropositivity (Table). Despite similar host species tested, our results only partially confirm observations of previous studies, where mainly stone martens and foxes were found to be seropositive in about 1 third of the animals tested (*6*). That discrepancy indicates the relevance of factors other than host susceptibility and host occurrence.

Univariable regression analysis (Figure) revealed that foxes from the Island Rügen collected close to the bay coast, close to the Baltic Sea, or close to watercourses had an increased risk for H5-specific antibodies (p<0.05) (Appendix Table 2, 3). Seropositivity increased with age; 11.6% (10/86) of the juvenile and 23.5% (43/183) of the adult foxes were positive for H5-specific antibodies. A final multivariable model (Appendix Table 4) selected by stepwise forward–backward selection, which also included age, revealed that, with increasing distance to watercourses (e.g., flowing waters, water source areas, streams, ditches, rivers, and canals), the risk for foxes being positive for H5-specific antibodies decreased (p<0.0001); however, with increasing distance to shrubland (e.g., hedges, bushes, field crops, groups of trees, rows of trees, avenues, dominant single trees, and groups of shrubs), the risk increased (p<0.005). In contrast, high proportions of shrubland in a 2.5-km buffer zone around the sampling site of foxes represented a protective factor (p<0.005), supporting the assumption of less exposure to HPAIV through water-associated hosts, including Anseriformes birds, for example, that are known to graze on open lands.

**Figure:**
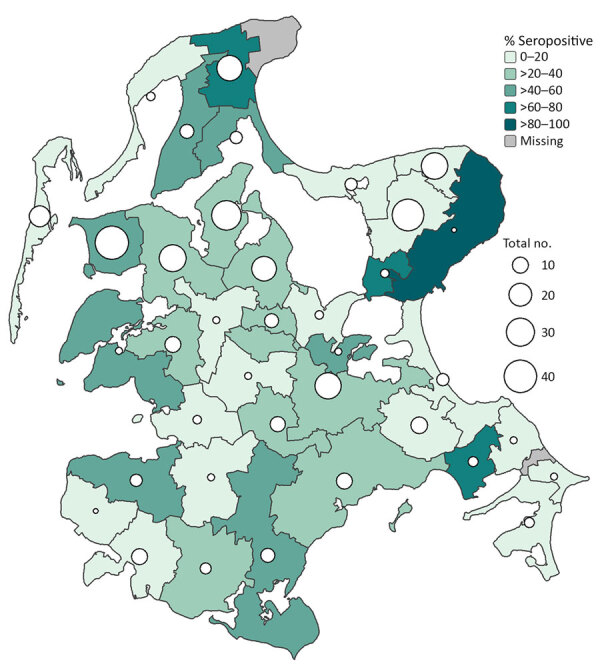
Percentage of highly pathogenic avian influenza subtype H5 seropositive juvenile and adult red foxes (*Vulpes vulpes*), by municipality, on the Island Rügen, northeast Germany, December 2023–February 2025. White circles represent total numbers of sampled and analyzed foxes per municipality.

The possible existence of a similar correlation for omnivorous species might serve as explanation for the 3.5% (95% CI 0.9%–8.7%) seropositivity in wild boar. In addition to their scavenging or predatory behavior (*8*), wild boars have been described as nest robbers of waterfowl species in other wetland areas in Europe (*9*). All individual animals with H5-specific antibodies belonged to family groups roaming in water-associated zones (e.g., the Island Rügen and Fischland-Darß-Zingst) (Appendix Table 1), where a higher likelihood of interaction with waterfowl species can be expected.

After the previous HPAIV H5N8 epizootic during 2016–2017, a broad serologic investigation in wild boars was conducted in southern Germany (*10*). Low-level prevalence of H5N8 neutralizing antibodies (1.13%) were detected; a single positive case occurred close to a prior HPAI outbreak in waterfowl (*10*,*11*). In domestic pigs the overall susceptibility in experimental settings remained low despite a high infectious dose of a single clade 2.3.4.4b strain (*12*); however, studies using different genotypes indicate variability in susceptibility, particularly in the extent of seroconversion (*13*,*14*).

Although little is known about the persistence of AIV antibodies in wild mammals, serologic surveillance might provide a more complete picture of past exposure to potentially infected avian (reservoir) hosts than molecular testing alone. The discrepancy in NP- and H5-antibody detection leaves the possibility of other AIV subtypes undergoing initial seroconversion, so a combination of testing approaches seems advisable.

## Conclusions

H5-specific antibodies in carnivores and wild Suidae species indicate ongoing spillover events, especially in wetland habitats potentially shared with main (reservoir) hosts (Anseriformes and Charadriiformes). The role of wild boars warrants further attention because Suidae species are considered mixing vessels for swine-, human-, and avian-derived IAVs (*15*). Every such infection in mammalian species provides opportunities for virus adaptations, so continuous surveillance is recommended. Consequently, continuous surveillance also should be considered for pet animals in hotspot areas (e.g., free-ranging cats or hunting dogs) as further potential bridges for transmitting infections from wildlife to private households.

Appendix Additional information about serologic surveillance of high pathogenicity avian influenza virus subtype H5 in wildlife, northeast Germany, 2023–2025.
