# Supplementary material for: Serologic Surveillance of Highly Pathogenic Avian Influenza Virus Subtype H5 in Wildlife, Northeast Germany, 2023–2025
Source: Emerg Infect Dis. 2026 May;32(5):790–3. doi: 10.3201/eid3205.251555 (PMC13175092; doi:10.3201/eid3205.251555)
Supplement: Appendix — Additional information about serologic surveillance of high pathogenicity avian influenza virus subtype H5 in wildlife, northeast Germany, 2023–2025. [file 25-1555-Techapp-s1.pdf]

Article DOI: https://doi.org/10.3201/eid3205.251555

EID cannot ensure accessibility for supplementary materials supplied by authors. Readers who have difficulty accessing supplementary content should contact the authors for assistance.

Serologic Surveillance of High Pathogenicity Avian Influenza Virus Subtype H5 in Wildlife, Northeast Germany, 2023–2025

Appendix

Methods

Sample collection

Samples have been collected during hunts in the field (swab samples from ruminants and wild boar) or during necropsies performed at the Friedrich-Loeffler-Institut (FLI) after a freeze-thaw cycle as described in Kunisch, et al. (*1*) (swabs and organ samples from carnivores). The number of sampled wildlife animals as well as their origin are available in the Appendix Table 1. One dry swab per individual was taken from both nostrils of predator and hoofed game species and immediately has been stored in 2 mL Minimum Essential Medium (MEM). Brain and lung samples from carnivorous species were collected during necropsy and transferred into tubes, prepared with 1.5 mL MEM and a 5 mm steel bead for homogenization in a TissueLyser (Qiagen, Hilden, Germany).

Samples for serological analyses were collected in form of whole blood or serum samples taken directly after the hunt out of the exit wounds (hoofed game) or from the body cavity of thawed carcasses during necropsy (carnivores; see 3. Laboratory Analyses).

The collected samples were stored at -20 °C until analyses.

Ethics Approval

All samples have been retrieved from animals that were hunted by local hunters according to the appropriate German legislation or that were found dead. No ethical approval is

required for this kind of sample collection in the federal state of Mecklenburg-Western Pomerania, Germany.

Laboratory Analyses

Swab sample fluid and organ homogenate supernatant (100 µL) served as input for bead-based extraction of nucleic acids on a King Fisher 96 Flex (Thermo Fisher Scientific, Braunschweig, Germany) with the NucleoMag® VET kit (Macherey-Nagel, Düren, Germany) following the manufacturer’s instructions.

The nucleic acid was subsequently tested by an RT-qPCR targeting influenza A virus (IAV) matrix (M) gene as described by Hoffmann, et al. (*2*) in combination with an internal control system based on the beta-actin gene (*3*).

The serum or plasma-like supernatant was applied to two consecutive commercial enzyme-linked immunosorbent assays (ELISA) according to the manufacturer's instructions. Samples of carnivorous species were collected from the body cavities of previously frozen carcasses. The thawing process in preparation for post mortem investigations, may have resulted in a dilution of the coagulated blood. Consequently, the protocol for "red body fluids" or "serum-like samples" from carnivores was adapted to utilize the same initial 1:5 dilution as the other samples, rather than adhering to the manufacturer's instructions for an 1:10 dilution. The ID Screen® Influenza A Antibody Competition Multi-species (Innovative Diagnostics, Grabels, France) allows for the detection of antibodies against the nucleoprotein (NP) of IAV. In case of positive or questionable results, those samples have been tested in the ID Screen® Influenza H5 Antibody Competition ELISA (Innovative Diagnostics, Grabels, France) to confirm the presence or absence of antibodies specific against hemagglutinin of subtype H5. Serological samples of carnivores and omnivores examined in this study were likewise used in Aebischer, et al. (A. Aebischer A et al., unpub. data, https://doi.org/10.64898/2026.01.05.697617) for the “*Development of a multi-species luciferase-based double antigen ELISA for the detection of antibodies against Influenza A virus H5 clade 2.3.4.4b*”, thus providing additional verification of our screening results.

In our study, the samples have been applied to an in-house serum neutralization assay (SNT) in an exemplary manner. In preparation, blood samples have been heat inactivated at 56 °C for 30 minutes. Due to the highly hemolytic nature of the blood samples, the sample

suitability for heat inactivation was severely compromised and only allowed for a very limited subset finally being tested via SNT (carnivore-panel n=12; wild boar samples n=6). We followed the procedure described by Hennig, et al. (*4*) with the following modifications: A trypsin-independent and highly pathogenic avian influenza virus was applied (HPAIV H5 strain *A/chicken/Ger-NI/4286/2022*) for the incubation, before a suspension of MDCK II cells (no. 0606; Collection of Cell Lines in Veterinary Medicine (CCLV), FLI, Greifswald - Insel Riems, Germany) in cell culture medium ($2x10^5$/mL) was added to each well. The strain *A/chicken/Ger-NI/4286/2022* has been classified as genotype AB ("EA-2021-AB"), representing one of the major genotypes circulating in Europe since 2021 and, thus, in the time span before sample collection (*5,6*).

The applied negative and positive control sera and the additional virus back-titration were tested in quadruplicates, so were the samples with few exceptions that lacked sufficient volume. The dilution cell culture medium for all steps was stocked with Baytril® (Enrofloxacine; 2.5 µg/mL; Elanco GmbH, Cuxhaven, Germany) and 5% fetal calf serum.

Spatial analysis

Methods and data used for the determination of landcover proportions and distance measurements

Statistical spatial analysis largely followed a procedure previously described (*7*). Coordinates of each animal capture site, spatial data on land cover in the entire area of interest and data on individual animals (e.g. age) were statistically assessed. A circular buffer zone of 2.5 km radius around the location where each animal carcass was found was used to approximates the home range of individual animals. The proportion of each landcover type in this buffer zone was calculated. Further, the distance of each animal location to the nearest polygon of a certain landcover type was calculated. Proportions, distances and individual animal data were used in logistic regression to model H5-specific seropositivity.

Data on land cover was retrieved from "Biotoptypen- und Nutzungstypenkartierung" (BNTK) provided by the federal state of Mecklenburg-Western Pomerania (last updated in 2012). Because the urban landscape of the Island Rügen (study area for foxes, racoon dogs and racoons) has expanded, replacing natural areas during the last two decades, BNTK data was combined with data from the "Digitale Basis-Landschaftsmodell" (DLM) provided by the

"Bundesamt für Kartografie und Geodäsie", increasing accuracy and better reflecting the actual situation.

Methods used for spatial analyses and other statistical tests

To identify environmental risk factors for H5-specific seropositivity, the proportion of each land cover type falling in the respective buffer zone for each animal was calculated using the sf package in R. In R, the distance of each animal to its nearest landcover of the specified landcover types was calculated using the st_nn function. Each of the distances was scaled to 100 m steps and buffer proportions to 10 % steps.

Differences in H5-specific seropositivity by sex and age were tested using Chi-square tests. The number of foxes, racoon dogs and racoons submitted and the proportion of animals testing positive were tabulated by the municipality and visualized in a choropleth map. Municipality boundaries were chosen for visualization purposes. As hunters are submitting animals voluntarily, the distribution of animals submitted per hunter was skewed, with a median of three animals per hunter, but as many as 80 or as few as one animal.

Univariable logistic regression was conducted for all distance and buffer variables of each landscape type, and if the P-value was < 0.2, the variable was included in a multivariable model. This threshold was established given the large number of variables in this dataset, aiming to only include those with potential relevance for H5 influenza infection in the multivariable model. The multivariable model was reduced by using forward-backwards selection in R, and the model with the lowest Akaike's Information Criterion (AIC; Akaike, 1974) is reported as final model. Additionally, the principle of Occam's razor was used when selecting the final variables, which states that when the difference in AIC between including a variable in a model is ≤ 2 compared to a model without it, the simpler model (meaning without the variable in question) is the better fit. A weighted Moran's I and Geary's C were run on the standardized deviance residuals of each univariable logistic regression model to check for underlying spatial autocorrelation. If following these tests, autocorrelation would be apparent, a spatial logistic regression model would be used that allows for accounting for the spatial clustering present (8). The goodness of fit of the reduced multivariable model was evaluated using the omnibus test and pseudo R^2.

Results of analysis

Results in foxes: Foxes represent the most frequently sampled carnivorous species with background information on their location of origin available. Regression analysis using environmental variables (i.e. either proportions in a buffer zone of 2.5 km radius or nearest distances to a particular environmental characteristic; Appendix Table 2) revealed a number of statistically significant associations, i.e. one at the level of $P < 0.01$ (Balticsea_dist100) and five at the level of $P \geq 0.01$ and $P < 0.05$ (Shrubland_prop, Urban_prop, Watercourses_prop, Baycoast_dist100, Watercourses_dist100). For the remaining variables displayed in Appendix Table 3 values of $P \geq 0.05$ and $P < 0.2$ had been calculated. All of these variables were included into a forward-backward selection process to identify the optimal final multivariable model. The initial analysis of putative associations of H5-specific antibodies with sex or age revealed that in adult foxes there was a statistically higher proportion of H5-specific seropositivity as compared to juvenile foxes Therefore, this variable was also included into the modelling to not disregard potential effects of age.

Results in raccoon dogs and racoons: Less data points were available for these animal species and same analyses as for foxes were performed. However, in this dataset, spatial autocorrelation was present, mandating the use of different analysis methods moving forward. Thus, results of these analyses are not displayed.

References

1. Kunisch AM, Wassermann J, Peters M, Feldmann K, Prinzenberg E, Klink JC, et al. In situ collected small intestinal mucosal swabs as alternative analytes to diagnose *Echinococcus multilocularis* infection in its definitive hosts by real-time polymerase chain reaction. Vet Parasitol. 2025;335:110418. PubMed https://doi.org/10.1016/j.vetpar.2025.110418

2. Hoffmann B, Harder T, Lange E, Kalthoff D, Reimann I, Grund C, et al. New real-time reverse transcriptase polymerase chain reactions facilitate detection and differentiation of novel A/H1N1 influenza virus in porcine and human samples. Berl Munch Tierarztl Wochenschr. 2010;123:286–92. PubMed

3. Toussaint JF, Sailleau C, Breard E, Zientara S, De Clercq K. Bluetongue virus detection by two real-time RT-qPCRs targeting two different genomic segments. J Virol Methods. 2007;140:115–23. PubMed https://doi.org/10.1016/j.jviromet.2006.11.007

4. Hennig C, Graaf-Rau A, Schmies K, Elling R, Henneke P, Dürrwald R, et al. High serological barriers may contribute to restricted influenza-A-virus transmission between pigs and humans. One Health. 2025;21:101214. https://doi.org/10.1016/j.onehlt.2025.101214

5. Piesche R, Breithaupt A, Pohlmann A, Ahrens AK, Beer M, Harder T, et al. Dominant HPAIV H5N1 genotypes of Germany 2021/2022 are linked to high virulence in Pekin ducklings. Npj Viruses. 2024;2:53. PubMed https://doi.org/10.1038/s44298-024-00062-0

6. Fusaro A, Zecchin B, Giussani E, Palumbo E, Agüero-García M, Bachofen C, et al. High pathogenic avian influenza A(H5) viruses of clade 2.3.4.4b in Europe—why trends of virus evolution are more difficult to predict. Virus Evol. 2024;10:veae027. PubMed https://doi.org/10.1093/ve/veae027

7. Rogoll L, Schulz K, Staubach C, Oļševskis E, Seržants M, Lamberga K, et al. Identification of predilection sites for wild boar carcass search based on spatial analysis of Latvian ASF surveillance data. Sci Rep. 2024;14:382. PubMed https://doi.org/10.1038/s41598-023-50477-7

Appendix Table 1. Overview on metadata for the analyzed samples, including sampling ID, group designation, (scientific) species name and sample origin. The results of enzyme linked immunosorbent assay (ELISA) approaches targeting influenza A virus' (IAV) nucleoprotein (NP) or hemagglutinin of subtype H5 and the reverse transcription quantitative real-time polymerase chain reaction (RT-qPCR) screening results for IAV ribonucleic acid (RNA) are presented, if available. For samples, not tested in a specific test, the respective columns are filled with "-".

Sample ID	Group	Species	Scientific species name	Sample origin rural district	Result IAV-NP antibody ELISA in percent*	Assessment IAV-NP antibody ELISA	Result IAV-H5 antibody ELISA in percent†	Assessment IAV-H5 antibody ELISA	Titer serum neutralization assay	IAV RT-qPCR nasal swab	IAV RT-qPCR lung	IAV RT-qPCR brain
2024BVD16887	Herbivore	European fallow deer	*Dama dama*	Vorpommern-Rügen	87	negative	-	-	-	negative	-	-
2024BVD16889	Herbivore	European fallow deer	*Dama dama*	Vorpommern-Rügen	107	negative	-	-	-	negative	-	-
2024BVD16890	Herbivore	European fallow deer	*Dama dama*	Vorpommern-Rügen	90	negative	-	-	-	negative	-	-
2024BVD16891	Herbivore	European fallow deer	*Dama dama*	Vorpommern-Rügen	123	negative	-	-	-	negative	-	-
2024BVD16892	Herbivore	European fallow deer	*Dama dama*	Vorpommern-Rügen	116	negative	-	-	-	negative	-	-
2024BVD16893	Herbivore	European fallow deer	*Dama dama*	Vorpommern-Rügen	119	negative	-	-	-	negative	-	-
2024BVD16894	Omnivore	Wild boar	*Sus scrofa*	Vorpommern-Rügen	115	negative	-	-	-	negative	-	-
2024BVD16895	Herbivore	European fallow deer	*Dama dama*	Vorpommern-Rügen	113	negative	-	-	-	negative	-	-
2024BVD16896	Herbivore	European fallow deer	*Dama dama*	Vorpommern-Rügen	117	negative	-	-	-	negative	-	-
2024BVD16897	Herbivore	European fallow deer	*Dama dama*	Vorpommern-Rügen	121	negative	-	-	-	negative	-	-
2024BVD16898	Herbivore	European fallow deer	*Dama dama*	Vorpommern-Rügen	125	negative	-	-	-	negative	-	-
2024BVD16899	Herbivore	Roe deer	*Capreolus capreolus*	Vorpommern-Rügen	110	negative	-	-	-	negative	-	-
2024BVD16901	Herbivore	Roe deer	*Capreolus capreolus*	Vorpommern-Rügen	117	negative	-	-	-	negative	-	-
2024BVD16902	Herbivore	European fallow deer	*Dama dama*	Vorpommern-Rügen	111	negative	-	-	-	negative	-	-
2024BVD16903	Omnivore	Wild boar	*Sus scrofa*	Vorpommern-Rügen	106	negative	-	-	-	negative	-	-
2024BVD16904	Herbivore	European fallow deer	*Dama dama*	Vorpommern-Rügen	112	negative	-	-	-	negative	-	-
2024BVD16905	Herbivore	European fallow deer	*Dama dama*	Vorpommern-Rügen	78	negative	-	-	-	negative	-	-
2024BVD16906	Herbivore	European fallow deer	*Dama dama*	Vorpommern-Rügen	100	negative	-	-	-	negative	-	-
2024BVD16907	Herbivore	European fallow deer	*Dama dama*	Vorpommern-Rügen	111	negative	-	-	-	negative	-	-
2024BVD16908	Herbivore	European fallow deer	*Dama dama*	Vorpommern-Rügen	122	negative	-	-	-	negative	-	-
2024BVD16909	Herbivore	European fallow deer	*Dama dama*	Vorpommern-Rügen	106	negative	-	-	-	negative	-	-
2024BVD16910	Herbivore	European fallow deer	*Dama dama*	Vorpommern-Rügen	106	negative	-	-	-	negative	-	-

Sample ID	Group	Species	Scientific species name	Sample origin rural district	Result IAV-NP antibody ELISA in percent*	Assessment IAV-NP antibody ELISA	Result IAV-H5 antibody ELISA in percent†	Assessment IAV-H5 antibody ELISA	Titer serum neutralization assay	IAV RT-qPCR nasal swab	IAV RT-qPCR lung	IAV RT-qPCR brain
2024BVD16911	Herbivore	European fallow deer	*Dama dama*	Vorpommern-Rügen	100	negative	-	-	-	negative	-	-
2024BVD16913	Herbivore	European fallow deer	*Dama dama*	Vorpommern-Rügen	115	negative	-	-	-	negative	-	-
2024BVD16914	Herbivore	European fallow deer	*Dama dama*	Vorpommern-Rügen	102	negative	-	-	-	negative	-	-
2024BVD16915	Herbivore	European fallow deer	*Dama dama*	Vorpommern-Rügen	112	negative	-	-	-	negative	-	-
2024BVD16916	Herbivore	European fallow deer	*Dama dama*	Vorpommern-Rügen	108	negative	-	-	-	negative	-	-
2024BVD16917	Herbivore	European fallow deer	*Dama dama*	Vorpommern-Rügen	110	negative	-	-	-	negative	-	-
2024BVD16918	Herbivore	European fallow deer	*Dama dama*	Vorpommern-Rügen	105	negative	-	-	-	negative	-	-
2024BVD16919	Herbivore	European fallow deer	*Dama dama*	Vorpommern-Rügen	103	negative	-	-	-	negative	-	-
2024BVD16920	Herbivore	European fallow deer	*Dama dama*	Vorpommern-Rügen	112	negative	-	-	-	negative	-	-
2024BVD16921	Herbivore	European fallow deer	*Dama dama*	Vorpommern-Rügen	114	negative	-	-	-	negative	-	-
2024BVD16922	Omnivore	Wild boar	*Sus scrofa*	Vorpommern-Rügen	100	negative	-	-	-	negative	-	-
2024BVD16923	Herbivore	Roe deer	*Capreolus capreolus*	Vorpommern-Rügen	97	negative	-	-	-	negative	-	-
2024BVD16924	Herbivore	Roe deer	*Capreolus capreolus*	Vorpommern-Rügen	98	negative	-	-	-	negative	-	-
2024BVD16925	Herbivore	Mouflon	*Ovis gmelini musimon*	Vorpommern-Rügen	112	negative	-	-	-	negative	-	-
2024BVD16926	Herbivore	Red deer	*Cervus elaphus*	Vorpommern-Rügen	107	negative	-	-	-	negative	-	-
2024BVD16927	Herbivore	Red deer	*Cervus elaphus*	Vorpommern-Rügen	106	negative	-	-	-	negative	-	-
2024BVD16928	Omnivore	Wild boar	*Sus scrofa*	Vorpommern-Rügen	107	negative	-	-	-	negative	-	-
2024BVD16929	Herbivore	Red deer	*Cervus elaphus*	Vorpommern-Rügen	112	negative	-	-	-	negative	-	-
2024BVD16930	Omnivore	Wild boar	*Sus scrofa*	Vorpommern-Rügen	125	negative	-	-	-	negative	-	-
2024BVD16931	Herbivore	Red deer	*Cervus elaphus*	Vorpommern-Rügen	107	negative	-	-	-	negative	-	-
2024BVD16932	Herbivore	Roe deer	*Capreolus capreolus*	Vorpommern-Rügen	105	negative	-	-	-	negative	-	-
2024BVD16934	Omnivore	Wild boar	*Sus scrofa*	Vorpommern-Rügen	23	positive	33	positive	<1:20	negative	-	-
2024BVD16935	Omnivore	Wild boar	*Sus scrofa*	Vorpommern-Rügen	102	negative	-	-	-	negative	-	-
2024BVD16936	Omnivore	Wild boar	*Sus scrofa*	Vorpommern-Rügen	111	negative	-	-	-	negative	-	-
2024BVD16937	Herbivore	Red deer	*Cervus elaphus*	Vorpommern-Rügen	109	negative	-	-	-	negative	-	-
2024BVD16938	Herbivore	Red deer	*Cervus elaphus*	Vorpommern-Rügen	102	negative	-	-	-	negative	-	-
2024BVD16939	Omnivore	Wild boar	*Sus scrofa*	Vorpommern-Rügen	69	negative	-	-	-	negative	-	-

Sample ID	Group	Species	Scientific species name	Sample origin rural district	Result IAV-NP antibody ELISA in percent*	Assessment IAV-NP antibody ELISA	Result IAV-H5 antibody ELISA in percent†	Assessment IAV-H5 antibody ELISA	Titer serum neutralization assay	IAV RT-qPCR nasal swab	IAV RT-qPCR lung	IAV RT-qPCR brain
2024BVD16940	Omnivore	Wild boar	*Sus scrofa*	Vorpommern-Rügen	109	negative	-	-	-	negative	-	-
2024BVD16941	Omnivore	Wild boar	*Sus scrofa*	Vorpommern-Rügen	116	negative	-	-	-	negative	-	-
2024BVD16942	Omnivore	Wild boar	*Sus scrofa*	Vorpommern-Rügen	99	negative	-	-	-	negative	-	-
2024BVD16943	Herbivore	Red deer	*Cervus elaphus*	Vorpommern-Rügen	110	negative	-	-	-	negative	-	-
2024BVD16944	Herbivore	Red deer	*Cervus elaphus*	Vorpommern-Rügen	105	negative	-	-	-	negative	-	-
2024BVD16945	Herbivore	Red deer	*Cervus elaphus*	Vorpommern-Rügen	106	negative	-	-	-	negative	-	-
2024BVD16946	Herbivore	Roe deer	*Capreolus capreolus*	Vorpommern-Rügen	109	negative	-	-	-	negative	-	-
2024BVD16947	Omnivore	Wild boar	*Sus scrofa*	Vorpommern-Rügen	112	negative	-	-	-	negative	-	-
2024BVD16948	Herbivore	Red deer	*Cervus elaphus*	Vorpommern-Rügen	105	negative	-	-	-	negative	-	-
2024BVD16949	Herbivore	Roe deer	*Capreolus capreolus*	Vorpommern-Rügen	99	negative	-	-	-	negative	-	-
2024BVD16950	Herbivore	Red deer	*Cervus elaphus*	Vorpommern-Rügen	105	negative	-	-	-	negative	-	-
2024BVD16951	Herbivore	Red deer	*Cervus elaphus*	Vorpommern-Rügen	107	negative	-	-	-	negative	-	-
2024BVD16952	Herbivore	Red deer	*Cervus elaphus*	Vorpommern-Rügen	119	negative	-	-	-	negative	-	-
2024BVD16953	Herbivore	Red deer	*Cervus elaphus*	Vorpommern-Rügen	108	negative	-	-	-	negative	-	-
2024BVD16954	Omnivore	Wild boar	*Sus scrofa*	Vorpommern-Rügen	107	negative	-	-	-	negative	-	-
2024BVD16955	Omnivore	Wild boar	*Sus scrofa*	Vorpommern-Rügen	100	negative	-	-	-	negative	-	-
2024BVD16956	Omnivore	Wild boar	*Sus scrofa*	Vorpommern-Rügen	114	negative	-	-	-	negative	-	-
2024BVD16957	Omnivore	Wild boar	*Sus scrofa*	Vorpommern-Rügen	95	negative	-	-	-	negative	-	-
2024BVD16958	Herbivore	Red deer	*Cervus elaphus*	Vorpommern-Rügen	110	negative	-	-	-	negative	-	-
2024BVD16959	Omnivore	Wild boar	*Sus scrofa*	Vorpommern-Rügen	99	negative	-	-	-	negative	-	-
2024BVD16960	Omnivore	Wild boar	*Sus scrofa*	Vorpommern-Rügen	111	negative	-	-	-	negative	-	-
2024BVD16961	Omnivore	Wild boar	*Sus scrofa*	Vorpommern-Rügen	109	negative	-	-	-	negative	-	-
2024BVD16962	Omnivore	Wild boar	*Sus scrofa*	Vorpommern-Rügen	111	negative	-	-	-	negative	-	-
2024BVD16963	Omnivore	Wild boar	*Sus scrofa*	Vorpommern-Rügen	27	positive	17	positive	<1:20	negative	-	-
2024BVD16964	Herbivore	Roe deer	*Capreolus capreolus*	Mecklenburgische Seenplatte	97	negative	-	-	-	negative	-	-
2024BVD16965	Omnivore	Wild boar	*Sus scrofa*	Mecklenburgische Seenplatte	114	negative	-	-	-	negative	-	-
2024BVD16966	Omnivore	Wild boar	*Sus scrofa*	Mecklenburgische Seenplatte	107	negative	-	-	-	negative	-	-
2024BVD17016	Herbivore	Roe deer	*Capreolus capreolus*	Vorpommern-Rügen	107	negative	-	-	-	negative	-	-
2024BVD17017	Herbivore	European fallow deer	*Dama dama*	Vorpommern-Rügen	113	negative	-	-	-	negative	-	-
2024BVD17018	Herbivore	Roe deer	*Capreolus capreolus*	Vorpommern-Rügen	115	negative	-	-	-	negative	-	-

Sample ID	Group	Species	Scientific species name	Sample origin rural district	Result IAV-NP antibody ELISA in percent*	Assessment IAV-NP antibody ELISA	Result IAV-H5 antibody ELISA in percent†	Assessment IAV-H5 antibody ELISA	Titer serum neutralization assay	IAV RT-qPCR nasal swab	IAV RT-qPCR lung	IAV RT-qPCR brain
2024BVD17019	Herbivore	European fallow deer	*Dama dama*	Vorpommern-Rügen	109	negative	-	-	-	negative	-	-
2024BVD17021	Herbivore	European fallow deer	*Dama dama*	Vorpommern-Rügen	106	negative	-	-	-	negative	-	-
2024BVD17022	Herbivore	European fallow deer	*Dama dama*	Vorpommern-Rügen	97	negative	-	-	-	negative	-	-
2024BVD17023	Herbivore	European fallow deer	*Dama dama*	Vorpommern-Rügen	116	negative	-	-	-	negative	-	-
2024BVD17024	Herbivore	European fallow deer	*Dama dama*	Vorpommern-Rügen	116	negative	-	-	-	negative	-	-
2024BVD17025	Herbivore	Roe deer	*Capreolus capreolus*	Vorpommern-Rügen	117	negative	-	-	-	negative	-	-
2024BVD17026	Herbivore	Roe deer	*Capreolus capreolus*	Vorpommern-Rügen	68	negative	-	-	-	negative	-	-
2024BVD17027	Herbivore	European fallow deer	*Dama dama*	Vorpommern-Rügen	76	negative	-	-	-	negative	-	-
2024BVD17028	Herbivore	European fallow deer	*Dama dama*	Vorpommern-Rügen	108	negative	-	-	-	negative	-	-
2024BVD17029	Omnivore	Wild boar	*Sus scrofa*	Vorpommern-Rügen	96	negative	-	-	-	negative	-	-
2024BVD17030	Omnivore	Wild boar	*Sus scrofa*	Vorpommern-Rügen	41	positive	100	negative	<1:20	negative	-	-
2024BVD17031	Omnivore	Wild boar	*Sus scrofa*	Vorpommern-Rügen	94	negative	-	-	-	negative	-	-
2024BVD17032	Omnivore	Wild boar	*Sus scrofa*	Vorpommern-Rügen	134	negative	-	-	-	negative	-	-
2024BVD17033	Omnivore	Wild boar	*Sus scrofa*	Vorpommern-Rügen	94	negative	-	-	-	negative	-	-
2024BVD17034	Herbivore	European fallow deer	*Dama dama*	Vorpommern-Rügen	96	negative	-	-	-	negative	-	-
2024BVD17035	Herbivore	Red deer	*Cervus elaphus*	Vorpommern-Rügen	90	negative	-	-	-	negative	-	-
2024BVD17036	Herbivore	European fallow deer	*Dama dama*	Vorpommern-Rügen	78	negative	-	-	-	negative	-	-
2024BVD17037	Herbivore	European fallow deer	*Dama dama*	Vorpommern-Rügen	97	negative	-	-	-	negative	-	-
2024BVD17038	Herbivore	European fallow deer	*Dama dama*	Vorpommern-Rügen	94	negative	-	-	-	negative	-	-
2024BVD17039	Herbivore	European fallow deer	*Dama dama*	Vorpommern-Rügen	97	negative	-	-	-	negative	-	-
2024BVD17040	Herbivore	Roe deer	*Capreolus capreolus*	Vorpommern-Rügen	94	negative	-	-	-	negative	-	-
2024BVD17041	Herbivore	European fallow deer	*Dama dama*	Vorpommern-Rügen	89	negative	-	-	-	negative	-	-
2024BVD17042	Herbivore	Roe deer	*Capreolus capreolus*	Vorpommern-Rügen	92	negative	-	-	-	negative	-	-
2024BVD17043	Herbivore	European fallow deer	*Dama dama*	Vorpommern-Rügen	87	negative	-	-	-	negative	-	-

Sample ID	Group	Species	Scientific species name	Sample origin rural district	Result IAV-NP antibody ELISA in percent*	Assessment IAV-NP antibody ELISA	Result IAV-H5 antibody ELISA in percent†	Assessment IAV-H5 antibody ELISA	Titer serum neutralization assay	IAV RT-qPCR nasal swab	IAV RT-qPCR lung	IAV RT-qPCR brain
2024BVD17044	Herbivore	European fallow deer	*Dama dama*	Vorpommern-Rügen	91	negative	-	-	-	negative	-	-
2024BVD17045	Omnivore	Wild boar	*Sus scrofa*	Vorpommern-Rügen	97	negative	-	-	-	negative	-	-
2024BVD17046	Omnivore	Wild boar	*Sus scrofa*	Vorpommern-Rügen	94	negative	-	-	-	negative	-	-
2024BVD17047	Omnivore	Wild boar	*Sus scrofa*	Vorpommern-Rügen	99	negative	-	-	-	negative	-	-
2024BVD17048	Omnivore	Wild boar	*Sus scrofa*	Vorpommern-Rügen	98	negative	-	-	-	negative	-	-
2024BVD17049	Omnivore	Wild boar	*Sus scrofa*	Vorpommern-Rügen	93	negative	-	-	-	negative	-	-
2024BVD17050	Omnivore	Wild boar	*Sus scrofa*	Vorpommern-Rügen	92	negative	-	-	-	negative	-	-
2024BVD17051	Omnivore	Wild boar	*Sus scrofa*	Vorpommern-Rügen	94	negative	-	-	-	negative	-	-
2024BVD17052	Herbivore	European fallow deer	*Dama dama*	Vorpommern-Rügen	96	negative	-	-	-	negative	-	-
2024BVD17053	Omnivore	Wild boar	*Sus scrofa*	Vorpommern-Rügen	93	negative	-	-	-	negative	-	-
2024BVD17055	Omnivore	Wild boar	*Sus scrofa*	Vorpommern-Rügen	103	negative	-	-	-	negative	-	-
2024BVD17056	Herbivore	European fallow deer	*Dama dama*	Vorpommern-Rügen	79	negative	-	-	-	negative	-	-
2024BVD17058	Herbivore	European fallow deer	*Dama dama*	Vorpommern-Rügen	94	negative	-	-	-	negative	-	-
2024BVD17059	Omnivore	Wild boar	*Sus scrofa*	Vorpommern-Rügen	92	negative	-	-	-	negative	-	-
2024BVD17060	Omnivore	Wild boar	*Sus scrofa*	Vorpommern-Rügen	94	negative	-	-	-	negative	-	-
2024BVD17061	Omnivore	Wild boar	*Sus scrofa*	Vorpommern-Rügen	96	negative	-	-	-	negative	-	-
2024BVD17062	Omnivore	Wild boar	*Sus scrofa*	Vorpommern-Rügen	70	negative	-	-	-	negative	-	-
2024BVD17063	Omnivore	Wild boar	*Sus scrofa*	Vorpommern-Rügen	103	negative	-	-	-	negative	-	-
2024BVD17064	Omnivore	Wild boar	*Sus scrofa*	Vorpommern-Rügen	103	negative	-	-	-	negative	-	-
2024BVD17065	Omnivore	Wild boar	*Sus scrofa*	Vorpommern-Rügen	121	negative	-	-	-	negative	-	-
2024BVD17066	Omnivore	Wild boar	*Sus scrofa*	Vorpommern-Rügen	95	negative	-	-	-	negative	-	-
2024BVD17067	Omnivore	Wild boar	*Sus scrofa*	Vorpommern-Rügen	87	negative	-	-	-	negative	-	-
2024BVD17160	Omnivore	Wild boar	*Sus scrofa*	Vorpommern-Greifswald	93	negative	-	-	-	negative	-	-
2024BVD17161	Herbivore	Roe deer	*Capreolus capreolus*	Vorpommern-Greifswald	97	negative	-	-	-	negative	-	-
2024BVD17162	Omnivore	Wild boar	*Sus scrofa*	Vorpommern-Greifswald	88	negative	-	-	-	negative	-	-
2024BVD17164	Omnivore	Wild boar	*Sus scrofa*	Vorpommern-Greifswald	95	negative	-	-	-	negative	-	-
2024BVD17165	Herbivore	Roe deer	*Capreolus capreolus*	Vorpommern-Greifswald	64	negative	-	-	-	negative	-	-
2024BVD17166	Herbivore	Roe deer	*Capreolus capreolus*	Vorpommern-Greifswald	98	negative	-	-	-	negative	-	-
2024BVD17167	Omnivore	Wild boar	*Sus scrofa*	Vorpommern-Greifswald	93	negative	-	-	-	negative	-	-

Sample ID	Group	Species	Scientific species name	Sample origin rural district	Result IAV-NP antibody ELISA in percent*	Assessment IAV-NP antibody ELISA	Result IAV-H5 antibody ELISA in percent†	Assessment IAV-H5 antibody ELISA	Titer serum neutralization assay	IAV RT-qPCR nasal swab	IAV RT-qPCR lung	IAV RT-qPCR brain
2024BVD17168	Herbivore	Roe deer	*Capreolus capreolus*	Vorpommern-Greifswald	86	negative	-	-	-	negative	-	-
2024BVD17169	Herbivore	Roe deer	*Capreolus capreolus*	Vorpommern-Greifswald	100	negative	-	-	-	negative	-	-
2024BVD17170	Omnivore	Wild boar	*Sus scrofa*	Vorpommern-Greifswald	97	negative	-	-	-	negative	-	-
2024BVD17171	Omnivore	Wild boar	*Sus scrofa*	Vorpommern-Greifswald	86	negative	-	-	-	negative	-	-
2024BVD17172	Omnivore	Wild boar	*Sus scrofa*	Vorpommern-Greifswald	97	negative	-	-	-	negative	-	-
2024BVD17173	Omnivore	Wild boar	*Sus scrofa*	Vorpommern-Greifswald	95	negative	-	-	-	negative	-	-
2024BVD17174	Herbivore	Roe deer	*Capreolus capreolus*	Vorpommern-Greifswald	92	negative	-	-	-	negative	-	-
2024BVD17175	Omnivore	Wild boar	*Sus scrofa*	Vorpommern-Greifswald	93	negative	-	-	-	negative	-	-
2024BVD17176	Omnivore	Wild boar	*Sus scrofa*	Vorpommern-Greifswald	69	negative	-	-	-	negative	-	-
2024BVD17177	Omnivore	Wild boar	*Sus scrofa*	Vorpommern-Greifswald	95	negative	-	-	-	negative	-	-
2024BVD17178	Herbivore	Roe deer	*Capreolus capreolus*	Vorpommern-Greifswald	95	negative	-	-	-	negative	-	-
2024BVD17179	Herbivore	Roe deer	*Capreolus capreolus*	Vorpommern-Greifswald	89	negative	-	-	-	negative	-	-
2024BVD17180	Omnivore	Wild boar	*Sus scrofa*	Vorpommern-Greifswald	92	negative	-	-	-	negative	-	-
2024BVD17181	Omnivore	Wild boar	*Sus scrofa*	Vorpommern-Greifswald	90	negative	-	-	-	negative	-	-
2024BVD17183	Omnivore	Wild boar	*Sus scrofa*	Vorpommern-Greifswald	95	negative	-	-	-	negative	-	-
2024BVD17184	Herbivore	Roe deer	*Capreolus capreolus*	Vorpommern-Greifswald	70	negative	-	-	-	negative	-	-
2024BVD17185	Herbivore	Roe deer	*Capreolus capreolus*	Vorpommern-Greifswald	98	negative	-	-	-	negative	-	-
2024BVD17186	Herbivore	Roe deer	*Capreolus capreolus*	Vorpommern-Greifswald	92	negative	-	-	-	negative	-	-
2024BVD17187	Omnivore	Wild boar	*Sus scrofa*	Vorpommern-Greifswald	96	negative	-	-	-	negative	-	-
2024BVD17188	Omnivore	Wild boar	*Sus scrofa*	Vorpommern-Greifswald	87	negative	-	-	-	negative	-	-
2024BVD17189	Omnivore	Wild boar	*Sus scrofa*	Vorpommern-Greifswald	86	negative	-	-	-	negative	-	-
2024BVD17190	Omnivore	Wild boar	*Sus scrofa*	Vorpommern-Greifswald	87	negative	-	-	-	negative	-	-

Sample ID	Group	Species	Scientific species name	Sample origin rural district	Result IAV-NP antibody ELISA in percent*	Assessment IAV-NP antibody ELISA	Result IAV-H5 antibody ELISA in percent†	Assessment IAV-H5 antibody ELISA	Titer serum neutralization assay	IAV RT-qPCR nasal swab	IAV RT-qPCR lung	IAV RT-qPCR brain
2024BVD17191	Herbivore	European fallow deer	*Dama dama*	Mecklenburgische Seenplatte	95	negative	-	-	-	negative	-	-
2024BVD17192	Omnivore	Wild boar	*Sus scrofa*	Mecklenburgische Seenplatte	95	negative	-	-	-	negative	-	-
2024BVD17193	Omnivore	Wild boar	*Sus scrofa*	Mecklenburgische Seenplatte	96	negative	-	-	-	negative	-	-
2024BVD17194	Herbivore	Roe deer	*Capreolus capreolus*	Mecklenburgische Seenplatte	89	negative	-	-	-	negative	-	-
2024BVD17195	Herbivore	Roe deer	*Capreolus capreolus*	Mecklenburgische Seenplatte	102	negative	-	-	-	negative	-	-
2024BVD17196	Herbivore	European fallow deer	*Dama dama*	Mecklenburgische Seenplatte	92	negative	-	-	-	negative	-	-
2024BVD17197	Herbivore	Roe deer	*Capreolus capreolus*	Mecklenburgische Seenplatte	97	negative	-	-	-	negative	-	-
2024BVD17198	Herbivore	Roe deer	*Capreolus capreolus*	Mecklenburgische Seenplatte	95	negative	-	-	-	negative	-	-
2024BVD17199	Herbivore	Roe deer	*Capreolus capreolus*	Mecklenburgische Seenplatte	100	negative	-	-	-	negative	-	-
2024BVD17200	Omnivore	Wild boar	*Sus scrofa*	Mecklenburgische Seenplatte	93	negative	-	-	-	negative	-	-
2024BVD17201	Herbivore	Roe deer	*Capreolus capreolus*	Mecklenburgische Seenplatte	78	negative	-	-	-	negative	-	-
2024BVD17202	Herbivore	Roe deer	*Capreolus capreolus*	Mecklenburgische Seenplatte	101	negative	-	-	-	negative	-	-
2024BVD17203	Herbivore	Roe deer	*Capreolus capreolus*	Mecklenburgische Seenplatte	97	negative	-	-	-	negative	-	-
2024BVD17204	Herbivore	Roe deer	*Capreolus capreolus*	Mecklenburgische Seenplatte	106	negative	-	-	-	negative	-	-
2024BVD17205	Herbivore	European fallow deer	*Dama dama*	Mecklenburgische Seenplatte	104	negative	-	-	-	negative	-	-
2024BVD17206	Omnivore	Wild boar	*Sus scrofa*	Mecklenburgische Seenplatte	104	negative	-	-	-	negative	-	-
2024BVD17208	Omnivore	Wild boar	*Sus scrofa*	Mecklenburgische Seenplatte	91	negative	-	-	-	negative	-	-
2024BVD17209	Herbivore	Roe deer	*Capreolus capreolus*	Mecklenburgische Seenplatte	96	negative	-	-	-	negative	-	-
2024BVD17210	Herbivore	European fallow deer	*Dama dama*	Mecklenburgische Seenplatte	93	negative	-	-	-	negative	-	-
2024BVD17211	Omnivore	Wild boar	*Sus scrofa*	Mecklenburgische Seenplatte	106	negative	-	-	-	negative	-	-
2024BVD17212	Omnivore	Wild boar	*Sus scrofa*	Mecklenburgische Seenplatte	99	negative	-	-	-	negative	-	-
2024BVD17213	Omnivore	Wild boar	*Sus scrofa*	Mecklenburgische Seenplatte	89	negative	-	-	-	negative	-	-

Sample ID	Group	Species	Scientific species name	Sample origin rural district	Result IAV-NP antibody ELISA in percent*	Assessment IAV-NP antibody ELISA	Result IAV-H5 antibody ELISA in percent†	Assessment IAV-H5 antibody ELISA	Titer serum neutralization assay	IAV RT-qPCR nasal swab	IAV RT-qPCR lung	IAV RT-qPCR brain
2024BVD17214	Omnivore	Wild boar	*Sus scrofa*	Mecklenburgische Seenplatte	96	negative	-	-	-	negative	-	-
2024BVD17215	Omnivore	Wild boar	*Sus scrofa*	Mecklenburgische Seenplatte	87	negative	-	-	-	negative	-	-
2024BVD17216	Omnivore	Wild boar	*Sus scrofa*	Mecklenburgische Seenplatte	100	negative	-	-	-	negative	-	-
2024BVD17217	Herbivore	European fallow deer	*Dama dama*	Mecklenburgische Seenplatte	78	negative	-	-	-	negative	-	-
2024BVD17218	Omnivore	Wild boar	*Sus scrofa*	Mecklenburgische Seenplatte	99	negative	-	-	-	negative	-	-
2024BVD17219	Herbivore	Roe deer	*Capreolus capreolus*	Mecklenburgische Seenplatte	96	negative	-	-	-	negative	-	-
2024BVD17220	Omnivore	Wild boar	*Sus scrofa*	Mecklenburgische Seenplatte	98	negative	-	-	-	negative	-	-
2024BVD17221	Omnivore	Wild boar	*Sus scrofa*	Mecklenburgische Seenplatte	87	negative	-	-	-	negative	-	-
2024BVD17222	Omnivore	Wild boar	*Sus scrofa*	Mecklenburgische Seenplatte	105	negative	-	-	-	negative	-	-
2024BVD17223	Herbivore	unknown	unknown	Mecklenburgische Seenplatte	87	negative	-	-	-	negative	-	-
2024BVD17224	Herbivore	Roe deer	*Capreolus capreolus*	Mecklenburgische Seenplatte	77	negative	-	-	-	negative	-	-
2024BVD17225	Herbivore	Roe deer	*Capreolus capreolus*	Mecklenburgische Seenplatte	106	negative	-	-	-	negative	-	-
2024BVD17226	Herbivore	Roe deer	*Capreolus capreolus*	Mecklenburgische Seenplatte	103	negative	-	-	-	negative	-	-
2024BVD17227	Herbivore	Roe deer	*Capreolus capreolus*	Mecklenburgische Seenplatte	100	negative	-	-	-	negative	-	-
2024BVD17228	Herbivore	Roe deer	*Capreolus capreolus*	Mecklenburgische Seenplatte	95	negative	-	-	-	negative	-	-
2024BVD17229	Herbivore	Roe deer	*Capreolus capreolus*	Mecklenburgische Seenplatte	79	negative	-	-	-	negative	-	-
2024BVD17230	Herbivore	Roe deer	*Capreolus capreolus*	Mecklenburgische Seenplatte	73	negative	-	-	-	negative	-	-
2024BVD17231	Herbivore	Roe deer	*Capreolus capreolus*	Mecklenburgische Seenplatte	86	negative	-	-	-	negative	-	-
2024BVD17232	Herbivore	European fallow deer	*Dama dama*	Mecklenburgische Seenplatte	111	negative	-	-	-	negative	-	-
2024BVD17233	Herbivore	Roe deer	*Capreolus capreolus*	Mecklenburgische Seenplatte	86	negative	-	-	-	negative	-	-
2024BVD17234	Herbivore	Roe deer	*Capreolus capreolus*	Mecklenburgische Seenplatte	75	negative	-	-	-	negative	-	-
2024BVD17235	Herbivore	Roe deer	*Capreolus capreolus*	Mecklenburgische Seenplatte	96	negative	-	-	-	negative	-	-

Sample ID	Group	Species	Scientific species name	Sample origin rural district	Result IAV-NP antibody ELISA in percent*	Assessment IAV-NP antibody ELISA	Result IAV-H5 antibody ELISA in percent†	Assessment IAV-H5 antibody ELISA	Titer serum neutralization assay	IAV RT-qPCR nasal swab	IAV RT-qPCR lung	IAV RT-qPCR brain
2024BVD17236	Herbivore	Roe deer	*Capreolus capreolus*	Mecklenburgische Seenplatte	91	negative	-	-	-	negative	-	-
2024BVD17237	Herbivore	Roe deer	*Capreolus capreolus*	Mecklenburgische Seenplatte	77	negative	-	-	-	negative	-	-
2024BVD17238	Herbivore	European fallow deer	*Dama dama*	Mecklenburgische Seenplatte	92	negative	-	-	-	negative	-	-
2024BVD17239	Herbivore	European fallow deer	*Dama dama*	Mecklenburgische Seenplatte	91	negative	-	-	-	negative	-	-
2024BVD17240	Herbivore	Roe deer	*Capreolus capreolus*	Mecklenburgische Seenplatte	95	negative	-	-	-	negative	-	-
2024BVD17241	Herbivore	Roe deer	*Capreolus capreolus*	Mecklenburgische Seenplatte	104	negative	-	-	-	negative	-	-
2024BVD17242	Herbivore	Roe deer	*Capreolus capreolus*	Mecklenburgische Seenplatte	103	negative	-	-	-	negative	-	-
2024BVD17243	Herbivore	Roe deer	*Capreolus capreolus*	Mecklenburgische Seenplatte	99	negative	-	-	-	negative	-	-
2024BVD17244	Herbivore	Roe deer	*Capreolus capreolus*	Mecklenburgische Seenplatte	105	negative	-	-	-	negative	-	-
2024BVD17245	Herbivore	Roe deer	*Capreolus capreolus*	Mecklenburgische Seenplatte	103	negative	-	-	-	negative	-	-
2024BVD17246	Herbivore	Roe deer	*Capreolus capreolus*	Mecklenburgische Seenplatte	97	negative	-	-	-	negative	-	-
2024BVD17247	Herbivore	Roe deer	*Capreolus capreolus*	Mecklenburgische Seenplatte	103	negative	-	-	-	negative	-	-
2024BVD17248	Herbivore	Roe deer	*Capreolus capreolus*	Mecklenburgische Seenplatte	93	negative	-	-	-	negative	-	-
2024BVD17249	Herbivore	Roe deer	*Capreolus capreolus*	Mecklenburgische Seenplatte	86	negative	-	-	-	negative	-	-
2024BVD17257	Herbivore	European fallow deer	*Dama dama*	Mecklenburgische Seenplatte	86	negative	-	-	-	negative	-	-
2024BVD17258	Herbivore	Roe deer	*Capreolus capreolus*	Mecklenburgische Seenplatte	75	negative	-	-	-	negative	-	-
2024BVD17259	Herbivore	Roe deer	*Capreolus capreolus*	Mecklenburgische Seenplatte	96	negative	-	-	-	negative	-	-
2024BVD17260	Herbivore	Roe deer	*Capreolus capreolus*	Mecklenburgische Seenplatte	91	negative	-	-	-	negative	-	-
2024BVD17261	Herbivore	Roe deer	*Capreolus capreolus*	Mecklenburgische Seenplatte	77	negative	-	-	-	negative	-	-
2024BVD17262	Herbivore	Roe deer	*Capreolus capreolus*	Mecklenburgische Seenplatte	92	negative	-	-	-	negative	-	-
2024BVD17263	Herbivore	Roe deer	*Capreolus capreolus*	Mecklenburgische Seenplatte	91	negative	-	-	-	negative	-	-
2024BVD17264	Herbivore	Roe deer	*Capreolus capreolus*	Mecklenburgische Seenplatte	95	negative	-	-	-	negative	-	-

Sample ID	Group	Species	Scientific species name	Sample origin rural district	Result IAV-NP antibody ELISA in percent*	Assessment IAV-NP antibody ELISA	Result IAV-H5 antibody ELISA in percent†	Assessment IAV-H5 antibody ELISA	Titer serum neutralization assay	IAV RT-qPCR nasal swab	IAV RT-qPCR lung	IAV RT-qPCR brain
2024BVD17265	Herbivore	Roe deer	*Capreolus capreolus*	Mecklenburgische Seenplatte	104	negative	-	-	-	negative	-	-
2024BVD17266	Herbivore	Roe deer	*Capreolus capreolus*	Mecklenburgische Seenplatte	103	negative	-	-	-	negative	-	-
2024BVD17267	Herbivore	Roe deer	*Capreolus capreolus*	Mecklenburgische Seenplatte	99	negative	-	-	-	negative	-	-
2024BVD17268	Herbivore	Roe deer	*Capreolus capreolus*	Mecklenburgische Seenplatte	105	negative	-	-	-	negative	-	-
2024BVD17269	Herbivore	Roe deer	*Capreolus capreolus*	Mecklenburgische Seenplatte	103	negative	-	-	-	negative	-	-
2024BVD17270	Herbivore	Roe deer	*Capreolus capreolus*	Mecklenburgische Seenplatte	97	negative	-	-	-	negative	-	-
2024BVD17271	Herbivore	Roe deer	*Capreolus capreolus*	Mecklenburgische Seenplatte	103	negative	-	-	-	negative	-	-
2024BVD17272	Herbivore	Roe deer	*Capreolus capreolus*	Mecklenburgische Seenplatte	93	negative	-	-	-	negative	-	-
2024BVD17273	Herbivore	Roe deer	*Capreolus capreolus*	Mecklenburgische Seenplatte	86	negative	-	-	-	negative	-	-
2024BVD17274	Herbivore	Roe deer	*Capreolus capreolus*	Mecklenburgische Seenplatte	106	negative	-	-	-	negative	-	-
2024BVD17275	Herbivore	Roe deer	*Capreolus capreolus*	Mecklenburgische Seenplatte	107	negative	-	-	-	negative	-	-
2024BVD17276	Herbivore	European fallow deer	*Dama dama*	Mecklenburgische Seenplatte	97	negative	-	-	-	negative	-	-
2024BVD17277	Herbivore	Roe deer	*Capreolus capreolus*	Mecklenburgische Seenplatte	105	negative	-	-	-	negative	-	-
2024BVD17278	Omnivore	Wild boar	*Sus scrofa*	Mecklenburgische Seenplatte	110	negative	-	-	-	negative	-	-
2024BVD17279	Herbivore	Roe deer	*Capreolus capreolus*	Mecklenburgische Seenplatte	93	negative	-	-	-	negative	-	-
2024BVD17280	Omnivore	Wild boar	*Sus scrofa*	Mecklenburgische Seenplatte	94	negative	-	-	-	negative	-	-
2024BVD17281	Omnivore	Wild boar	*Sus scrofa*	Mecklenburgische Seenplatte	97	negative	-	-	-	negative	-	-
2024BVD17282	Herbivore	Roe deer	*Capreolus capreolus*	Mecklenburgische Seenplatte	101	negative	-	-	-	negative	-	-
2024BVD17283	Omnivore	Wild boar	*Sus scrofa*	Mecklenburgische Seenplatte	106	negative	-	-	-	negative	-	-
2024BVD17284	Omnivore	Wild boar	*Sus scrofa*	Mecklenburgische Seenplatte	84	negative	-	-	-	negative	-	-
2024BVD17285	Omnivore	Wild boar	*Sus scrofa*	Mecklenburgische Seenplatte	110	negative	-	-	-	negative	-	-
2024BVD17286	Omnivore	Wild boar	*Sus scrofa*	Mecklenburgische Seenplatte	104	negative	-	-	-	negative	-	-

Sample ID	Group	Species	Scientific species name	Sample origin rural district	Result IAV-NP antibody ELISA in percent*	Assessment IAV-NP antibody ELISA	Result IAV-H5 antibody ELISA in percent†	Assessment IAV-H5 antibody ELISA	Titer serum neutralization assay	IAV RT-qPCR nasal swab	IAV RT-qPCR lung	IAV RT-qPCR brain
2024BVD17287	Omnivore	Wild boar	*Sus scrofa*	Mecklenburgische Seenplatte	106	negative	-	-	-	negative	-	-
2024BVD17288	Omnivore	Wild boar	*Sus scrofa*	Mecklenburgische Seenplatte	115	negative	-	-	-	negative	-	-
2024BVD17289	Omnivore	Wild boar	*Sus scrofa*	Mecklenburgische Seenplatte	107	negative	-	-	-	negative	-	-
2024BVD17369	Herbivore	European fallow deer	*Dama dama*	Vorpommern-Rügen	102	negative	-	-	-	negative	-	-
2024BVD17370	Herbivore	European fallow deer	*Dama dama*	Vorpommern-Rügen	114	negative	-	-	-	negative	-	-
2024BVD17371	Herbivore	European fallow deer	*Dama dama*	Vorpommern-Rügen	103	negative	-	-	-	negative	-	-
2024BVD17372	Herbivore	European fallow deer	*Dama dama*	Vorpommern-Rügen	81	negative	-	-	-	negative	-	-
2024BVD17373	Herbivore	European fallow deer	*Dama dama*	Vorpommern-Rügen	104	negative	-	-	-	negative	-	-
2024BVD17374	Herbivore	European fallow deer	*Dama dama*	Vorpommern-Rügen	102	negative	-	-	-	negative	-	-
2024BVD17375	Herbivore	Roe deer	*Capreolus capreolus*	Vorpommern-Rügen	109	negative	-	-	-	negative	-	-
2024BVD17376	Herbivore	Roe deer	*Capreolus capreolus*	Vorpommern-Rügen	107	negative	-	-	-	negative	-	-
2024BVD17377	Herbivore	European fallow deer	*Dama dama*	Vorpommern-Rügen	97	negative	-	-	-	negative	-	-
2024BVD17378	Herbivore	Roe deer	*Capreolus capreolus*	Vorpommern-Rügen	105	negative	-	-	-	negative	-	-
2024BVD17379	Herbivore	European fallow deer	*Dama dama*	Vorpommern-Rügen	102	negative	-	-	-	negative	-	-
2024BVD17380	Omnivore	Wild boar	*Sus scrofa*	Vorpommern-Rügen	104	negative	-	-	-	negative	-	-
2024BVD17381	Omnivore	Wild boar	*Sus scrofa*	Vorpommern-Rügen	107	negative	-	-	-	negative	-	-
2024BVD17382	Herbivore	European fallow deer	*Dama dama*	Vorpommern-Rügen	106	negative	-	-	-	negative	-	-
2024BVD17383	Herbivore	European fallow deer	*Dama dama*	Vorpommern-Rügen	110	negative	-	-	-	negative	-	-
2024BVD17467	Herbivore	European fallow deer	*Dama dama*	Vorpommern-Rügen	108	negative	-	-	-	negative	-	-
2024BVD17468	Herbivore	European fallow deer	*Dama dama*	Vorpommern-Rügen	97	negative	-	-	-	negative	-	-
2024BVD17469	Omnivore	Wild boar	*Sus scrofa*	Vorpommern-Rügen	100	negative	-	-	-	negative	-	-
2024BVD17470	Omnivore	Wild boar	*Sus scrofa*	Vorpommern-Rügen	103	negative	-	-	-	negative	-	-
2024BVD17471	Herbivore	European fallow deer	*Dama dama*	Vorpommern-Rügen	89	negative	-	-	-	negative	-	-
2024BVD17472	Herbivore	European fallow deer	*Dama dama*	Vorpommern-Rügen	88	negative	-	-	-	negative	-	-

Sample ID	Group	Species	Scientific species name	Sample origin rural district	Result IAV-NP antibody ELISA in percent*	Assessment IAV-NP antibody ELISA	Result IAV-H5 antibody ELISA in percent†	Assessment IAV-H5 antibody ELISA	Titer serum neutralization assay	IAV RT-qPCR nasal swab	IAV RT-qPCR lung	IAV RT-qPCR brain
2024BVD17473	Herbivore	European fallow deer	*Dama dama*	Vorpommern-Rügen	94	negative	-	-	-	negative	-	-
2024BVD17474	Herbivore	European fallow deer	*Dama dama*	Vorpommern-Rügen	94	negative	-	-	-	negative	-	-
2024BVD17475	Herbivore	European fallow deer	*Dama dama*	Vorpommern-Rügen	92	negative	-	-	-	negative	-	-
2024BVD17476	Herbivore	European fallow deer	*Dama dama*	Vorpommern-Rügen	89	negative	-	-	-	negative	-	-
2024BVD17477	Herbivore	European fallow deer	*Dama dama*	Vorpommern-Rügen	106	negative	-	-	-	negative	-	-
2024BVD17478	Omnivore	Wild boar	*Sus scrofa*	Vorpommern-Rügen	99	negative	-	-	-	negative	-	-
2024BVD17479	Herbivore	European fallow deer	*Dama dama*	Vorpommern-Rügen	95	negative	-	-	-	negative	-	-
2024BVD17480	Omnivore	Wild boar	*Sus scrofa*	Vorpommern-Rügen	85	negative	-	-	-	negative	-	-
2024BVD17481	Omnivore	Wild boar	*Sus scrofa*	Vorpommern-Rügen	42	positive	4	positive	1:20	negative	-	-
2024BVD17482	Omnivore	Wild boar	*Sus scrofa*	Vorpommern-Rügen	20	positive	5	positive	<1:20	negative	-	-
2024BVD17483	Herbivore	Roe deer	*Capreolus capreolus*	Vorpommern-Rügen	98	negative	-	-	-	negative	-	-
2024BVD17484	Herbivore	European fallow deer	*Dama dama*	Vorpommern-Rügen	89	negative	-	-	-	negative	-	-
2024BVD17485	Herbivore	European fallow deer	*Dama dama*	Vorpommern-Rügen	114	negative	-	-	-	negative	-	-
2024BVD17487	Omnivore	Wild boar	*Sus scrofa*	Vorpommern-Rügen	31	positive	53	undetermined	<1:20	negative	-	-
2024BVD17488	Herbivore	Roe deer	*Capreolus capreolus*	Vorpommern-Rügen	103	negative	-	-	-	negative	-	-
2024BVD17489	Herbivore	European fallow deer	*Dama dama*	Vorpommern-Rügen	101	negative	-	-	-	negative	-	-
2024BVD17490	Herbivore	European fallow deer	*Dama dama*	Vorpommern-Rügen	102	negative	-	-	-	negative	-	-
2024BVD17491	Herbivore	European fallow deer	*Dama dama*	Vorpommern-Rügen	99	negative	-	-	-	negative	-	-
2024BVD17492	Herbivore	European fallow deer	*Dama dama*	Vorpommern-Rügen	90	negative	-	-	-	negative	-	-
2024BVD17493	Herbivore	European fallow deer	*Dama dama*	Vorpommern-Rügen	103	negative	-	-	-	negative	-	-
2024BVD17494	Herbivore	European fallow deer	*Dama dama*	Vorpommern-Rügen	98	negative	-	-	-	negative	-	-
2024BVD17495	Herbivore	European fallow deer	*Dama dama*	Vorpommern-Rügen	102	negative	-	-	-	negative	-	-
2024BVD17496	Omnivore	Wild boar	*Sus scrofa*	Vorpommern-Rügen	100	negative	-	-	-	negative	-	-
2024BVD17497	Herbivore	European fallow deer	*Dama dama*	Vorpommern-Rügen	97	negative	-	-	-	negative	-	-
2024BVD17498	Herbivore	European fallow deer	*Dama dama*	Vorpommern-Rügen	104	negative	-	-	-	negative	-	-

Sample ID	Group	Species	Scientific species name	Sample origin rural district	Result IAV-NP antibody ELISA in percent*	Assessment IAV-NP antibody ELISA	Result IAV-H5 antibody ELISA in percent†	Assessment IAV-H5 antibody ELISA	Titer serum neutralization assay	IAV RT-qPCR nasal swab	IAV RT-qPCR lung	IAV RT-qPCR brain
2024BVD17499	Herbivore	European fallow deer	*Dama dama*	Vorpommern-Rügen	101	negative	-	-	-	negative	-	-
2024BVD17500	Herbivore	European fallow deer	*Dama dama*	Vorpommern-Rügen	98	negative	-	-	-	negative	-	-
2024BVD17501	Herbivore	European fallow deer	*Dama dama*	Vorpommern-Rügen	97	negative	-	-	-	negative	-	-
2024BVD17502	Omnivore	Wild boar	*Sus scrofa*	Vorpommern-Rügen	96	negative	-	-	-	negative	-	-
2024BVD17503	Omnivore	Wild boar	*Sus scrofa*	Vorpommern-Rügen	100	negative	-	-	-	negative	-	-
2024BVD17504	Herbivore	Red deer	*Cervus elaphus*	Vorpommern-Rügen	88	negative	-	-	-	negative	-	-
2024BVD17505	Herbivore	Red deer	*Cervus elaphus*	Vorpommern-Rügen	92	negative	-	-	-	negative	-	-
2024BVD17506	Herbivore	European fallow deer	*Dama dama*	Vorpommern-Rügen	100	negative	-	-	-	negative	-	-
2024BVD17507	Herbivore	European fallow deer	*Dama dama*	Vorpommern-Rügen	93	negative	-	-	-	negative	-	-
2024BVD17508	Herbivore	European fallow deer	*Dama dama*	Vorpommern-Rügen	92	negative	-	-	-	negative	-	-
2024BVD17509	Herbivore	European fallow deer	*Dama dama*	Vorpommern-Rügen	106	negative	-	-	-	negative	-	-
2024BVD17510	Herbivore	European fallow deer	*Dama dama*	Vorpommern-Rügen	104	negative	-	-	-	negative	-	-
2024BVD17511	Herbivore	European fallow deer	*Dama dama*	Vorpommern-Rügen	103	negative	-	-	-	negative	-	-
2024BVD17512	Herbivore	European fallow deer	*Dama dama*	Vorpommern-Rügen	101	negative	-	-	-	negative	-	-
2024BVD17513	Herbivore	European fallow deer	*Dama dama*	Vorpommern-Rügen	98	negative	-	-	-	negative	-	-
2024BVD17515	Herbivore	European fallow deer	*Dama dama*	Vorpommern-Rügen	102	negative	-	-	-	negative	-	-
2024BVD17516	Herbivore	European fallow deer	*Dama dama*	Vorpommern-Rügen	100	negative	-	-	-	negative	-	-
2024BVD17517	Herbivore	Roe deer	*Capreolus capreolus*	Vorpommern-Rügen	102	negative	-	-	-	negative	-	-
2024BVD17519	Herbivore	European fallow deer	*Dama dama*	Vorpommern-Rügen	102	negative	-	-	-	negative	-	-
2024BVD17520	Omnivore	Wild boar	*Sus scrofa*	Vorpommern-Rügen	95	negative	-	-	-	negative	-	-
2024BVD17521	Omnivore	Wild boar	*Sus scrofa*	Vorpommern-Rügen	94	negative	-	-	-	negative	-	-
2024BVD17522	Herbivore	Red deer	*Cervus elaphus*	Vorpommern-Rügen	98	negative	-	-	-	negative	-	-
2024BVD17523	Omnivore	Wild boar	*Sus scrofa*	Vorpommern-Rügen	100	negative	-	-	-	negative	-	-
2024BVD17524	Herbivore	European fallow deer	*Dama dama*	Vorpommern-Rügen	95	negative	-	-	-	negative	-	-
2024BVD17525	Herbivore	European fallow deer	*Dama dama*	Vorpommern-Rügen	100	negative	-	-	-	negative	-	-
2024BVD17526	Omnivore	Wild boar	*Sus scrofa*	Vorpommern-Rügen	97	negative	-	-	-	negative	-	-
2024BVD17527	Omnivore	Wild boar	*Sus scrofa*	Vorpommern-Rügen	97	negative	-	-	-	negative	-	-
2024BVD17528	Omnivore	Wild boar	*Sus scrofa*	Vorpommern-Rügen	97	negative	-	-	-	negative	-	-

Sample ID	Group	Species	Scientific species name	Sample origin rural district	Result IAV-NP antibody ELISA in percent*	Assessment IAV-NP antibody ELISA	Result IAV-H5 antibody ELISA in percent†	Assessment IAV-H5 antibody ELISA	Titer serum neutralization assay	IAV RT-qPCR nasal swab	IAV RT-qPCR lung	IAV RT-qPCR brain
2024BVD17529	Herbivore	Red deer	*Cervus elaphus*	Vorpommern-Rügen	97	negative	-	-	-	negative	-	-
2024BVD17530	Omnivore	Wild boar	*Sus scrofa*	Vorpommern-Rügen	102	negative	-	-	-	negative	-	-
2024BVD17531	Omnivore	Wild boar	*Sus scrofa*	Vorpommern-Rügen	98	negative	-	-	-	negative	-	-
2024BVD17532	Omnivore	Wild boar	*Sus scrofa*	Vorpommern-Rügen	92	negative	-	-	-	negative	-	-
2024BVD17533	Omnivore	Wild boar	*Sus scrofa*	Vorpommern-Rügen	102	negative	-	-	-	negative	-	-
2024BVD17534	Omnivore	Wild boar	*Sus scrofa*	Vorpommern-Rügen	93	negative	-	-	-	negative	-	-
2024BVD17535	Herbivore	Red deer	*Cervus elaphus*	Vorpommern-Rügen	98	negative	-	-	-	negative	-	-
2024BVD17536	Omnivore	Wild boar	*Sus scrofa*	Vorpommern-Rügen	94	negative	-	-	-	negative	-	-
2024BVD17537	Herbivore	Red deer	*Cervus elaphus*	Vorpommern-Rügen	89	negative	-	-	-	negative	-	-
2024BVD17538	Herbivore	Red deer	*Cervus elaphus*	Vorpommern-Rügen	95	negative	-	-	-	negative	-	-
2024BVD17539	Omnivore	Wild boar	*Sus scrofa*	Vorpommern-Rügen	94	negative	-	-	-	negative	-	-
2024BVD17542	Herbivore	Roe deer	*Capreolus capreolus*	Vorpommern-Greifswald	90	negative	-	-	-	negative	-	-
2024BVD17543	Herbivore	Roe deer	*Capreolus capreolus*	Vorpommern-Greifswald	86	negative	-	-	-	negative	-	-
2024BVD17544	Herbivore	European fallow deer	*Dama dama*	Vorpommern-Greifswald	96	negative	-	-	-	negative	-	-
2024BVD17545	Herbivore	Roe deer	*Capreolus capreolus*	Vorpommern-Greifswald	91	negative	-	-	-	negative	-	-
2024BVD17546	Herbivore	Roe deer	*Capreolus capreolus*	Vorpommern-Greifswald	92	negative	-	-	-	negative	-	-
2024BVD17547	Herbivore	Roe deer	*Capreolus capreolus*	Vorpommern-Greifswald	72	negative	-	-	-	negative	-	-
2024BVD17548	Herbivore	European fallow deer	*Dama dama*	Vorpommern-Greifswald	77	negative	-	-	-	negative	-	-
2025BVD00072	Herbivore	Mouflon	*Ovis gmelini musimon*	Vorpommern-Rügen	95	negative	-	-	-	not taken	-	-
2025BVD00073	Herbivore	Mouflon	*Ovis gmelini musimon*	Vorpommern-Rügen	96	negative	-	-	-	not taken	-	-
2025BVD00074	Herbivore	Mouflon	*Ovis gmelini musimon*	Vorpommern-Rügen	96	negative	-	-	-	not taken	-	-
2025BVD00075	Herbivore	Mouflon	*Ovis gmelini musimon*	Vorpommern-Rügen	94	negative	-	-	-	not taken	-	-
2025BVD00076	Herbivore	Mouflon	*Ovis gmelini musimon*	Vorpommern-Rügen	93	negative	-	-	-	not taken	-	-
2025BVD00846	Herbivore	Mouflon	*Ovis gmelini musimon*	Vorpommern-Rügen	96	negative	-	-	-	not taken	-	-
2023BVD08586	Carnivore	Red fox	*Vulpes vulpes*	Vorpommern-Rügen	26	positive	44	positive	-	negative	negative	negative
2023BVD08587	Carnivore	Red fox	*Vulpes vulpes*	Vorpommern-Rügen	97	negative	-	-	-	negative	negative	negative
2023BVD08588	Carnivore	Raccoon	*Procyon lotor*	Vorpommern-Rügen	104	negative	-	-	-	negative	negative	negative
2023BVD08589	Carnivore	Red fox	*Vulpes vulpes*	Vorpommern-Rügen	82	negative	-	-	-	negative	negative	negative
2023BVD08590	Carnivore	Red fox	*Vulpes vulpes*	Vorpommern-Rügen	48	undetermined	41	positive	-	negative	negative	negative
2023BVD08591	Carnivore	Red fox	*Vulpes vulpes*	Vorpommern-Rügen	29	positive	48	positive	-	negative	negative	negative
2023BVD08592	Carnivore	Red fox	*Vulpes vulpes*	Vorpommern-Rügen	67	negative	-	-	-	negative	negative	negative

Sample ID	Group	Species	Scientific species name	Sample origin rural district	Result IAV-NP antibody ELISA in percent*	Assessment IAV-NP antibody ELISA	Result IAV-H5 antibody ELISA in percent†	Assessment IAV-H5 antibody ELISA	Titer serum neutralization assay	IAV RT-qPCR nasal swab	IAV RT-qPCR lung	IAV RT-qPCR brain
2023BVD08593	Carnivore	Raccoon dog	*Nyctereutes procyonoides*	Vorpommern-Rügen	90	negative	-	-	-	negative	negative	negative
2023BVD08594	Carnivore	Red fox	*Vulpes vulpes*	Vorpommern-Rügen	98	negative	-	-	-	negative	negative	negative
2023BVD08595	Carnivore	Raccoon dog	*Nyctereutes procyonoides*	Vorpommern-Rügen	73	negative	-	-	-	negative	negative	negative
2023BVD08596	Carnivore	Raccoon dog	*Nyctereutes procyonoides*	Vorpommern-Rügen	86	negative	-	-	-	negative	negative	negative
2023BVD08597	Carnivore	Red fox	*Vulpes vulpes*	Vorpommern-Rügen	79	negative	-	-	-	negative	negative	negative
2023BVD08598	Carnivore	Red fox	*Vulpes vulpes*	Vorpommern-Rügen	80	negative	-	-	-	negative	negative	negative
2023BVD08599	Carnivore	Red fox	*Vulpes vulpes*	Vorpommern-Rügen	not taken	not taken	not taken	not taken	-	negative	negative	negative
2023BVD08600	Carnivore	Red fox	*Vulpes vulpes*	Vorpommern-Rügen	86	negative	-	-	-	negative	negative	negative
2023BVD08601	Carnivore	Raccoon	*Procyon lotor*	Vorpommern-Rügen	61	negative	-	-	-	negative	negative	negative
2023BVD08602	Carnivore	Raccoon dog	*Nyctereutes procyonoides*	Vorpommern-Rügen	41	positive	37	positive	-	negative	negative	negative
2023BVD08603	Carnivore	Red fox	*Vulpes vulpes*	Vorpommern-Rügen	74	negative	-	-	-	negative	negative	not taken
2023BVD08604	Carnivore	Red fox	*Vulpes vulpes*	Vorpommern-Rügen	90	negative	-	-	-	negative	negative	negative
2023BVD08605	Carnivore	Red fox	*Vulpes vulpes*	Vorpommern-Rügen	78	negative	-	-	-	negative	negative	negative
2023BVD08606	Carnivore	Red fox	*Vulpes vulpes*	Vorpommern-Rügen	86	negative	-	-	-	negative	negative	negative
2023BVD08607	Carnivore	Red fox	*Vulpes vulpes*	Vorpommern-Rügen	86	negative	-	-	-	negative	negative	negative
2023BVD08608	Carnivore	Raccoon	*Procyon lotor*	Vorpommern-Rügen	80	negative	-	-	-	negative	negative	negative
2023BVD08609	Carnivore	Raccoon dog	*Nyctereutes procyonoides*	Vorpommern-Rügen	113	negative	-	-	-	negative	negative	negative
2023BVD08610	Carnivore	Raccoon	*Procyon lotor*	Vorpommern-Rügen	92	negative	-	-	-	negative	negative	negative
2023BVD08611	Carnivore	Red fox	*Vulpes vulpes*	Vorpommern-Rügen	96	negative	-	-	-	negative	negative	negative
2023BVD08694	Carnivore	Red fox	*Vulpes vulpes*	Vorpommern-Rügen	97	negative	-	-	-	negative	negative	negative
2023BVD08695	Carnivore	Red fox	*Vulpes vulpes*	Vorpommern-Rügen	74	negative	-	-	-	negative	negative	negative
2023BVD08696	Carnivore	Red fox	*Vulpes vulpes*	Vorpommern-Rügen	89	negative	-	-	-	negative	negative	negative
2023BVD08697	Carnivore	Red fox	*Vulpes vulpes*	Vorpommern-Rügen	36	positive	not available	not available	-	negative	negative	negative
2023BVD08698	Carnivore	Raccoon	*Procyon lotor*	Vorpommern-Rügen	91	negative	-	-	-	negative	negative	negative
2023BVD08699	Carnivore	Red fox	*Vulpes vulpes*	Vorpommern-Rügen	99	negative	-	-	-	negative	negative	negative
2023BVD08700	Carnivore	Red fox	*Vulpes vulpes*	Vorpommern-Rügen	93	negative	-	-	-	negative	negative	negative
2023BVD08701	Carnivore	Red fox	*Vulpes vulpes*	Vorpommern-Rügen	72	negative	-	-	-	negative	negative	negative
2023BVD08702	Carnivore	Red fox	*Vulpes vulpes*	Vorpommern-Rügen	19	positive	7	positive	-	negative	negative	negative
2023BVD08703	Carnivore	Red fox	*Vulpes vulpes*	Vorpommern-Rügen	84	negative	-	-	-	negative	negative	negative
2023BVD08704	Carnivore	Red fox	*Vulpes vulpes*	Vorpommern-Rügen	91	negative	-	-	-	negative	negative	negative
2023BVD08705	Carnivore	Red fox	*Vulpes vulpes*	Vorpommern-Rügen	82	negative	-	-	-	negative	negative	negative
2023BVD08706	Carnivore	Red fox	*Vulpes vulpes*	Vorpommern-Rügen	85	negative	-	-	-	negative	negative	negative
2023BVD08707	Carnivore	Red fox	*Vulpes vulpes*	Vorpommern-Rügen	89	negative	-	-	-	negative	negative	negative
2023BVD08708	Carnivore	Red fox	*Vulpes vulpes*	Vorpommern-Rügen	38	positive	70	negative	-	negative	negative	negative
2023BVD09100	Carnivore	Red fox	*Vulpes vulpes*	Vorpommern-Rügen	23	positive	48	positive	-	negative	negative	negative
2024BVD00105	Carnivore	Raccoon dog	*Nyctereutes procyonoides*	Vorpommern-Rügen	90	negative	-	-	-	negative	negative	negative
2024BVD00106	Carnivore	Red fox	*Vulpes vulpes*	Vorpommern-Rügen	92	negative	-	-	-	negative	negative	negative

Sample ID	Group	Species	Scientific species name	Sample origin rural district	Result IAV-NP antibody ELISA in percent*	Assessment IAV-NP antibody ELISA	Result IAV-H5 antibody ELISA in percent†	Assessment IAV-H5 antibody ELISA	Titer serum neutralization assay	IAV RT-qPCR nasal swab	IAV RT-qPCR lung	IAV RT-qPCR brain
2024BVD00107	Carnivore	Raccoon dog	*Nyctereutes procyonoides*	Vorpommern-Rügen	84	negative	-	-	-	negative	negative	negative
2024BVD00108	Carnivore	Red fox	*Vulpes vulpes*	Vorpommern-Rügen	11	positive	11	positive	-	negative	negative	negative
2024BVD00109	Carnivore	Red fox	*Vulpes vulpes*	Vorpommern-Rügen	88	negative	-	-	-	negative	negative	negative
2024BVD00110	Carnivore	Red fox	*Vulpes vulpes*	Vorpommern-Rügen	64	negative	-	-	-	negative	negative	negative
2024BVD00111	Carnivore	Raccoon dog	*Nyctereutes procyonoides*	Vorpommern-Rügen	92	negative	-	-	-	negative	negative	negative
2024BVD00112	Carnivore	Red fox	*Vulpes vulpes*	Vorpommern-Rügen	90	negative	-	-	-	negative	negative	negative
2024BVD00113	Carnivore	Raccoon	*Procyon lotor*	Vorpommern-Rügen	96	negative	-	-	-	negative	negative	negative
2024BVD00114	Carnivore	Red fox	*Vulpes vulpes*	Vorpommern-Rügen	90	negative	-	-	-	negative	negative	negative
2024BVD00115	Carnivore	Raccoon	*Procyon lotor*	Vorpommern-Rügen	106	negative	-	-	-	negative	negative	negative
2024BVD00116	Carnivore	Red fox	*Vulpes vulpes*	Vorpommern-Rügen	89	negative	-	-	-	negative	negative	negative
2024BVD00117	Carnivore	Red fox	*Vulpes vulpes*	Vorpommern-Rügen	not available	not available	not available	not available	-	negative	negative	negative
2024BVD00118	Carnivore	Red fox	*Vulpes vulpes*	Vorpommern-Rügen	20	positive	41	positive	-	negative	negative	not taken
2024BVD00119	Carnivore	Red fox	*Vulpes vulpes*	Vorpommern-Rügen	89	negative	-	-	-	negative	negative	negative
2024BVD00120	Carnivore	Red fox	*Vulpes vulpes*	Vorpommern-Rügen	81	negative	-	-	-	negative	negative	negative
2024BVD00121	Carnivore	Red fox	*Vulpes vulpes*	Vorpommern-Rügen	81	negative	-	-	-	negative	negative	negative
2024BVD00122	Carnivore	Red fox	*Vulpes vulpes*	Vorpommern-Rügen	90	negative	-	-	-	negative	negative	negative
2024BVD00123	Carnivore	Raccoon dog	*Nyctereutes procyonoides*	Vorpommern-Rügen	109	negative	-	-	-	negative	negative	negative
2024BVD00124	Carnivore	Red fox	*Vulpes vulpes*	Vorpommern-Rügen	92	negative	-	-	-	negative	negative	negative
2024BVD00125	Carnivore	Red fox	*Vulpes vulpes*	Vorpommern-Rügen	59	negative	-	-	-	negative	negative	negative
2024BVD00127	Carnivore	Red fox	*Vulpes vulpes*	Vorpommern-Rügen	16	positive	8	positive	-	negative	negative	negative
2024BVD00128	Carnivore	Raccoon dog	*Nyctereutes procyonoides*	Vorpommern-Rügen	83	negative	-	-	-	negative	negative	negative
2024BVD00129	Carnivore	Red fox	*Vulpes vulpes*	Vorpommern-Rügen	95	negative	-	-	-	negative	negative	negative
2024BVD00130	Carnivore	Red fox	*Vulpes vulpes*	Vorpommern-Rügen	33	positive	5	positive	-	negative	negative	negative
2024BVD00131	Carnivore	Red fox	*Vulpes vulpes*	Vorpommern-Rügen	33	positive	67	negative	-	negative	negative	negative
2024BVD00132	Carnivore	Red fox	*Vulpes vulpes*	Vorpommern-Rügen	23	positive	5	positive	-	negative	negative	negative
2024BVD00133	Carnivore	Raccoon dog	*Nyctereutes procyonoides*	Vorpommern-Rügen	91	negative	-	-	-	negative	negative	negative
2024BVD00134	Carnivore	Raccoon dog	*Nyctereutes procyonoides*	Vorpommern-Rügen	74	negative	-	-	-	negative	negative	negative
2024BVD00135	Carnivore	Red fox	*Vulpes vulpes*	Vorpommern-Rügen	79	negative	-	-	-	negative	negative	negative
2024BVD00136	Carnivore	Raccoon dog	*Nyctereutes procyonoides*	Vorpommern-Rügen	35	positive	37	positive	-	negative	negative	not taken
2024BVD00137	Carnivore	Red fox	*Vulpes vulpes*	Vorpommern-Rügen	82	negative	-	-	-	negative	negative	negative
2024BVD00138	Carnivore	Raccoon dog	*Nyctereutes procyonoides*	Vorpommern-Rügen	87	negative	-	-	-	negative	negative	negative
2024BVD00139	Carnivore	Red fox	*Vulpes vulpes*	Vorpommern-Rügen	34	positive	26	positive	-	negative	negative	negative
2024BVD00140	Carnivore	Raccoon dog	*Nyctereutes procyonoides*	Vorpommern-Rügen	89	negative	-	-	-	negative	negative	negative
2024BVD00141	Carnivore	Red fox	*Vulpes vulpes*	Vorpommern-Rügen	48	undetermined	55	undetermined	-	negative	negative	negative
2024BVD00142	Carnivore	Red fox	*Vulpes vulpes*	Vorpommern-Rügen	17	positive	68	negative	-	negative	negative	negative

Sample ID	Group	Species	Scientific species name	Sample origin rural district	Result IAV-NP antibody ELISA in percent*	Assessment IAV-NP antibody ELISA	Result IAV-H5 antibody ELISA in percent†	Assessment IAV-H5 antibody ELISA	Titer serum neutralization assay	IAV RT-qPCR nasal swab	IAV RT-qPCR lung	IAV RT-qPCR brain
2024BVD00143	Carnivore	Red fox	*Vulpes vulpes*	Vorpommern-Rügen	36	positive	12	positive	-	negative	negative	negative
2024BVD00144	Carnivore	Red fox	*Vulpes vulpes*	Vorpommern-Rügen	98	negative	-	-	-	negative	negative	negative
2024BVD00145	Carnivore	Red fox	*Vulpes vulpes*	Vorpommern-Rügen	86	negative	-	-	-	negative	negative	negative
2024BVD00146	Carnivore	Red fox	*Vulpes vulpes*	Vorpommern-Rügen	94	negative	-	-	-	negative	negative	negative
2024BVD00147	Carnivore	Red fox	*Vulpes vulpes*	Vorpommern-Rügen	100	negative	-	-	-	negative	negative	negative
2024BVD00148	Carnivore	Red fox	*Vulpes vulpes*	Vorpommern-Rügen	11	positive	21	positive	-	not taken	negative	negative
2024BVD00149	Carnivore	Red fox	*Vulpes vulpes*	Vorpommern-Rügen	12	positive	14	positive	-	not taken	negative	negative
2024BVD00150	Carnivore	Red fox	*Vulpes vulpes*	Vorpommern-Rügen	105	negative	-	-	-	not taken	negative	negative
2024BVD00151	Carnivore	Red fox	*Vulpes vulpes*	Vorpommern-Rügen	106	negative	-	-	-	not taken	negative	negative
2024BVD02899	Carnivore	Red fox	*Vulpes vulpes*	Vorpommern-Rügen	10	positive	27	positive	-	negative	negative	negative
2024BVD02900	Carnivore	Raccoon	*Procyon lotor*	Vorpommern-Rügen	83	negative	-	-	-	negative	negative	negative
2024BVD02901	Carnivore	Red fox	*Vulpes vulpes*	Vorpommern-Rügen	81	negative	-	-	-	negative	negative	negative
2024BVD02902	Carnivore	Raccoon dog	*Nyctereutes procyonoides*	Vorpommern-Rügen	67	negative	-	-	-	negative	negative	negative
2024BVD02903	Carnivore	Red fox	*Vulpes vulpes*	Vorpommern-Rügen	40	positive	69	negative	-	negative	negative	negative
2024BVD02904	Carnivore	Red fox	*Vulpes vulpes*	Vorpommern-Rügen	5	positive	40	positive	-	negative	negative	negative
2024BVD02905	Carnivore	Raccoon dog	*Nyctereutes procyonoides*	Vorpommern-Rügen	not taken	not taken	not taken	not taken	-	negative	negative	negative
2024BVD02906	Carnivore	Red fox	*Vulpes vulpes*	Vorpommern-Rügen	90	negative	-	-	-	negative	negative	negative
2024BVD02907	Carnivore	Red fox	*Vulpes vulpes*	Vorpommern-Rügen	99	negative	-	-	-	negative	negative	negative
2024BVD02908	Carnivore	Red fox	*Vulpes vulpes*	Vorpommern-Rügen	107	negative	-	-	-	negative	negative	negative
2024BVD02909	Carnivore	Raccoon dog	*Nyctereutes procyonoides*	Vorpommern-Rügen	96	negative	-	-	-	negative	negative	negative
2024BVD02910	Carnivore	Red fox	*Vulpes vulpes*	Vorpommern-Rügen	10	positive	46	positive	1:40	negative	negative	negative
2024BVD02911	Carnivore	Raccoon dog	*Nyctereutes procyonoides*	Vorpommern-Rügen	87	negative	-	-	-	negative	negative	negative
2024BVD02912	Carnivore	Red fox	*Vulpes vulpes*	Vorpommern-Rügen	9	positive	6	positive	-	negative	negative	negative
2024BVD02913	Carnivore	Raccoon dog	*Nyctereutes procyonoides*	Vorpommern-Rügen	94	negative	-	-	-	negative	negative	negative
2024BVD02914	Carnivore	Red fox	*Vulpes vulpes*	Vorpommern-Rügen	not taken	not taken	not taken	not taken	-	negative	negative	negative
2024BVD02915	Carnivore	Red fox	*Vulpes vulpes*	Vorpommern-Rügen	73	negative	-	-	-	negative	negative	negative
2024BVD02916	Carnivore	Red fox	*Vulpes vulpes*	Vorpommern-Rügen	76	negative	-	-	-	negative	negative	negative
2024BVD02917	Carnivore	Red fox	*Vulpes vulpes*	Vorpommern-Rügen	68	negative	-	-	-	negative	negative	negative
2024BVD02918	Carnivore	Red fox	*Vulpes vulpes*	Vorpommern-Rügen	88	negative	-	-	-	negative	negative	negative
2024BVD02919	Carnivore	Red fox	*Vulpes vulpes*	Vorpommern-Rügen	10	positive	5	positive	-	negative	negative	negative
2024BVD02920	Carnivore	Red fox	*Vulpes vulpes*	Vorpommern-Rügen	94	negative	-	-	-	negative	negative	negative
2024BVD02921	Carnivore	Raccoon dog	*Nyctereutes procyonoides*	Vorpommern-Rügen	90	negative	-	-	-	negative	negative	negative
2024BVD02922	Carnivore	Red fox	*Vulpes vulpes*	Vorpommern-Rügen	88	negative	-	-	-	negative	negative	negative
2024BVD02923	Carnivore	Red fox	*Vulpes vulpes*	Vorpommern-Rügen	15	positive	31	positive	-	negative	negative	not taken
2024BVD03122	Carnivore	Raccoon dog	*Nyctereutes procyonoides*	Vorpommern-Rügen	92	negative	-	-	-	negative	negative	negative
2024BVD03123	Carnivore	Red fox	*Vulpes vulpes*	Vorpommern-Rügen	86	negative	-	-	-	negative	negative	negative
2024BVD03124	Carnivore	Red fox	*Vulpes vulpes*	Vorpommern-Rügen	7	positive	20	positive	-	negative	negative	negative

Sample ID	Group	Species	Scientific species name	Sample origin rural district	Result IAV-NP antibody ELISA in percent*	Assessment IAV-NP antibody ELISA	Result IAV-H5 antibody ELISA in percent†	Assessment IAV-H5 antibody ELISA	Titer serum neutralization assay	IAV RT-qPCR nasal swab	IAV RT-qPCR lung	IAV RT-qPCR brain
2024BVD03125	Carnivore	Red fox	*Vulpes vulpes*	Vorpommern-Rügen	86	negative	-	-	-	negative	negative	negative
2024BVD03126	Carnivore	Red fox	*Vulpes vulpes*	Vorpommern-Rügen	54	negative	-	-	-	negative	negative	negative
2024BVD03127	Carnivore	Raccoon	*Procyon lotor*	Vorpommern-Rügen	6	positive	41	positive	-	negative	negative	negative
2024BVD03128	Carnivore	Raccoon dog	*Nyctereutes procyonoides*	Vorpommern-Rügen	91	negative	-	-	-	negative	negative	negative
2024BVD03129	Carnivore	Red fox	*Vulpes vulpes*	Vorpommern-Rügen	91	negative	-	-	-	negative	negative	negative
2024BVD03130	Carnivore	Red fox	*Vulpes vulpes*	Vorpommern-Rügen	91	negative	-	-	-	negative	negative	negative
2024BVD03131	Carnivore	Red fox	*Vulpes vulpes*	Vorpommern-Rügen	83	negative	-	-	-	negative	negative	negative
2024BVD03132	Carnivore	Red fox	*Vulpes vulpes*	Vorpommern-Rügen	13	positive	7	positive	-	negative	negative	negative
2024BVD03133	Carnivore	Raccoon dog	*Nyctereutes procyonoides*	Vorpommern-Rügen	50	negative	-	-	-	negative	negative	negative
2024BVD03134	Carnivore	Red fox	*Vulpes vulpes*	Vorpommern-Rügen	60	negative	-	-	-	negative	negative	negative
2024BVD03135	Carnivore	Red fox	*Vulpes vulpes*	Vorpommern-Rügen	84	negative	-	-	-	negative	negative	negative
2024BVD03136	Carnivore	Red fox	*Vulpes vulpes*	Vorpommern-Rügen	91	negative	-	-	-	negative	negative	negative
2024BVD03137	Carnivore	Red fox	*Vulpes vulpes*	Vorpommern-Rügen	95	negative	-	-	-	negative	negative	negative
2024BVD03138	Carnivore	Red fox	*Vulpes vulpes*	Vorpommern-Rügen	92	negative	-	-	-	negative	negative	negative
2024BVD03139	Carnivore	Red fox	*Vulpes vulpes*	Vorpommern-Rügen	101	negative	-	-	-	negative	negative	negative
2024BVD03140	Carnivore	Red fox	*Vulpes vulpes*	Vorpommern-Rügen	94	negative	-	-	-	negative	negative	negative
2024BVD03141	Carnivore	European badger	*Meles meles*	Vorpommern-Rügen	80	negative	-	-	-	negative	negative	negative
2024BVD03142	Carnivore	Red fox	*Vulpes vulpes*	Vorpommern-Rügen	105	negative	-	-	-	negative	negative	negative
2024BVD03143	Carnivore	Red fox	*Vulpes vulpes*	Vorpommern-Rügen	80	negative	-	-	-	negative	negative	negative
2024BVD03144	Carnivore	Red fox	*Vulpes vulpes*	Vorpommern-Rügen	84	negative	-	-	-	negative	negative	negative
2024BVD03145	Carnivore	Red fox	*Vulpes vulpes*	Vorpommern-Rügen	6	positive	6	positive	-	negative	negative	negative
2024BVD03146	Carnivore	Raccoon dog	*Nyctereutes procyonoides*	Vorpommern-Rügen	100	negative	-	-	-	negative	negative	negative
2024BVD03207	Carnivore	Raccoon dog	*Nyctereutes procyonoides*	Vorpommern-Rügen	24	positive	29	positive	-	negative	negative	negative
2024BVD03208	Carnivore	Red fox	*Vulpes vulpes*	Vorpommern-Rügen	15	positive	7	positive	-	negative	negative	negative
2024BVD03209	Carnivore	Red fox	*Vulpes vulpes*	Vorpommern-Rügen	70	negative	-	-	-	negative	negative	negative
2024BVD03210	Carnivore	Red fox	*Vulpes vulpes*	Vorpommern-Rügen	99	negative	-	-	-	negative	negative	negative
2024BVD03211	Carnivore	Red fox	*Vulpes vulpes*	Vorpommern-Rügen	106	negative	-	-	-	negative	negative	negative
2024BVD03212	Carnivore	Red fox	*Vulpes vulpes*	Vorpommern-Rügen	88	negative	-	-	-	negative	negative	negative
2024BVD03213	Carnivore	Raccoon dog	*Nyctereutes procyonoides*	Vorpommern-Rügen	95	negative	-	-	-	negative	negative	negative
2024BVD03214	Carnivore	Red fox	*Vulpes vulpes*	Vorpommern-Rügen	91	negative	-	-	-	negative	negative	negative
2024BVD03215	Carnivore	Red fox	*Vulpes vulpes*	Vorpommern-Rügen	83	negative	-	-	-	negative	negative	negative
2024BVD03216	Carnivore	Red fox	*Vulpes vulpes*	Vorpommern-Rügen	85	negative	-	-	-	negative	negative	negative
2024BVD03217	Carnivore	Red fox	*Vulpes vulpes*	Vorpommern-Rügen	102	negative	-	-	-	negative	negative	negative
2024BVD03218	Carnivore	Red fox	*Vulpes vulpes*	Vorpommern-Rügen	86	negative	-	-	-	negative	negative	negative
2024BVD03219	Carnivore	Red fox	*Vulpes vulpes*	Vorpommern-Rügen	90	negative	-	-	-	negative	negative	negative
2024BVD03220	Carnivore	Red fox	*Vulpes vulpes*	Vorpommern-Rügen	107	negative	-	-	-	negative	negative	negative
2024BVD03221	Carnivore	Red fox	*Vulpes vulpes*	Vorpommern-Rügen	91	negative	-	-	-	negative	negative	negative
2024BVD03222	Carnivore	Red fox	*Vulpes vulpes*	Vorpommern-Rügen	49	undetermined	18	positive	-	negative	negative	negative

Sample ID	Group	Species	Scientific species name	Sample origin rural district	Result IAV-NP antibody ELISA in percent*	Assessment IAV-NP antibody ELISA	Result IAV-H5 antibody ELISA in percent†	Assessment IAV-H5 antibody ELISA	Titer serum neutralization assay	IAV RT-qPCR nasal swab	IAV RT-qPCR lung	IAV RT-qPCR brain
2024BVD03223	Carnivore	Red fox	*Vulpes vulpes*	Vorpommern-Rügen	94	negative	-	-	-	negative	negative	negative
2024BVD03224	Carnivore	Red fox	*Vulpes vulpes*	Vorpommern-Rügen	98	negative	-	-	-	negative	negative	negative
2024BVD03225	Carnivore	Red fox	*Vulpes vulpes*	Vorpommern-Rügen	101	negative	-	-	-	negative	negative	negative
2024BVD03226	Carnivore	Red fox	*Vulpes vulpes*	Vorpommern-Rügen	9	positive	48	positive	1:60	negative	negative	negative
2024BVD03227	Carnivore	Red fox	*Vulpes vulpes*	Vorpommern-Rügen	91	negative	-	-	-	negative	negative	negative
2024BVD03228	Carnivore	Red fox	*Vulpes vulpes*	Vorpommern-Rügen	86	negative	-	-	-	negative	negative	negative
2024BVD03229	Carnivore	Red fox	*Vulpes vulpes*	Vorpommern-Rügen	5	positive	27	positive	-	negative	negative	negative
2024BVD03230	Carnivore	Raccoon dog	*Nyctereutes procyonoides*	Vorpommern-Rügen	66	negative	-	-	-	negative	negative	negative
2024BVD03231	Carnivore	Raccoon	*Procyon lotor*	Vorpommern-Rügen	96	negative	-	-	-	negative	negative	negative
2024BVD03232	Carnivore	Red fox	*Vulpes vulpes*	Vorpommern-Rügen	97	negative	-	-	-	negative	negative	negative
2024BVD03233	Carnivore	Raccoon dog	*Nyctereutes procyonoides*	Vorpommern-Rügen	82	negative	-	-	-	negative	negative	negative
2024BVD03234	Carnivore	Red fox	*Vulpes vulpes*	Vorpommern-Rügen	45	undetermined	15	positive	-	negative	negative	negative
2024BVD03235	Carnivore	Raccoon	*Procyon lotor*	Vorpommern-Rügen	8	positive	36	positive	-	negative	negative	negative
2024BVD03236	Carnivore	Red fox	*Vulpes vulpes*	Vorpommern-Rügen	83	negative	-	-	-	negative	negative	negative
2024BVD04004	Carnivore	Raccoon dog	*Nyctereutes procyonoides*	Vorpommern-Rügen	32	positive	22	positive	-	negative	negative	negative
2024BVD04005	Carnivore	Red fox	*Vulpes vulpes*	Vorpommern-Rügen	115	negative	-	-	-	negative	negative	negative
2024BVD04006	Carnivore	Raccoon	*Procyon lotor*	Vorpommern-Rügen	8	positive	35	positive	-	negative	negative	negative
2024BVD04007	Carnivore	European badger	*Meles meles*	Vorpommern-Rügen	96	negative	-	-	-	negative	negative	negative
2024BVD04008	Carnivore	Raccoon dog	*Nyctereutes procyonoides*	Vorpommern-Rügen	13	positive	50	positive	-	negative	negative	negative
2024BVD04009	Carnivore	Raccoon dog	*Nyctereutes procyonoides*	Vorpommern-Rügen	81	negative	-	-	-	negative	negative	negative
2024BVD04010	Carnivore	Red fox	*Vulpes vulpes*	Vorpommern-Rügen	9	positive	12	positive	-	negative	negative	negative
2024BVD04011	Carnivore	Red fox	*Vulpes vulpes*	Vorpommern-Rügen	51	negative	-	-	-	negative	negative	negative
2024BVD04012	Carnivore	Red fox	*Vulpes vulpes*	Vorpommern-Rügen	97	negative	-	-	-	negative	negative	negative
2024BVD04013	Carnivore	Red fox	*Vulpes vulpes*	Vorpommern-Rügen	15	positive	5	positive	-	negative	negative	negative
2024BVD04014	Carnivore	Red fox	*Vulpes vulpes*	Vorpommern-Rügen	91	negative	-	-	-	negative	negative	negative
2024BVD04015	Carnivore	Red fox	*Vulpes vulpes*	Vorpommern-Rügen	85	negative	-	-	-	negative	negative	negative
2024BVD04016	Carnivore	Red fox	*Vulpes vulpes*	Vorpommern-Rügen	14	positive	48	positive	-	negative	negative	negative
2024BVD04017	Carnivore	Red fox	*Vulpes vulpes*	Vorpommern-Rügen	7	positive	13	positive	-	negative	negative	negative
2024BVD04018	Carnivore	Red fox	*Vulpes vulpes*	Vorpommern-Rügen	6	positive	22	positive	-	negative	negative	negative
2024BVD04019	Carnivore	Red fox	*Vulpes vulpes*	Vorpommern-Rügen	56	negative	-	-	-	negative	negative	negative
2024BVD04020	Carnivore	Raccoon	*Procyon lotor*	Vorpommern-Rügen	4	positive	32	positive	-	negative	negative	negative
2024BVD04021	Carnivore	Red fox	*Vulpes vulpes*	Vorpommern-Rügen	86	negative	-	-	-	negative	negative	negative
2024BVD04022	Carnivore	Raccoon dog	*Nyctereutes procyonoides*	Vorpommern-Rügen	84	negative	-	-	-	negative	negative	negative
2024BVD04023	Carnivore	Raccoon dog	*Nyctereutes procyonoides*	Vorpommern-Rügen	75	negative	-	-	-	negative	negative	negative
2024BVD04024	Carnivore	Red fox	*Vulpes vulpes*	Vorpommern-Rügen	not taken	not taken	not taken	not taken	-	negative	negative	negative
2024BVD04025	Carnivore	Red fox	*Vulpes vulpes*	Vorpommern-Rügen	94	negative	-	-	-	negative	negative	negative

Sample ID	Group	Species	Scientific species name	Sample origin rural district	Result IAV-NP antibody ELISA in percent*	Assessment IAV-NP antibody ELISA	Result IAV-H5 antibody ELISA in percent†	Assessment IAV-H5 antibody ELISA	Titer serum neutralization assay	IAV RT-qPCR nasal swab	IAV RT-qPCR lung	IAV RT-qPCR brain
2024BVD04026	Carnivore	Red fox	*Vulpes vulpes*	Vorpommern-Rügen	not taken	not taken	not taken	not taken	-	negative	negative	negative
2024BVD04027	Carnivore	Red fox	*Vulpes vulpes*	Vorpommern-Rügen	90	negative	-	-	-	negative	negative	negative
2024BVD04028	Carnivore	Red fox	*Vulpes vulpes*	Vorpommern-Rügen	not taken	not taken	not taken	not taken	-	negative	negative	negative
2024BVD04029	Carnivore	Red fox	*Vulpes vulpes*	Vorpommern-Rügen	88	negative	-	-	-	negative	negative	negative
2024BVD04030	Carnivore	Red fox	*Vulpes vulpes*	Vorpommern-Rügen	90	negative	-	-	-	negative	negative	negative
2024BVD04031	Carnivore	Red fox	*Vulpes vulpes*	Vorpommern-Rügen	29	positive	10	positive	-	negative	negative	negative
2024BVD04032	Carnivore	Red fox	*Vulpes vulpes*	Vorpommern-Rügen	not taken	not taken	not taken	not taken	-	negative	negative	negative
2024BVD04033	Carnivore	Red fox	*Vulpes vulpes*	Vorpommern-Rügen	89	negative	-	-	-	negative	negative	negative
2024BVD04034	Carnivore	Red fox	*Vulpes vulpes*	Vorpommern-Rügen	86	negative	-	-	-	negative	negative	negative
2024BVD04035	Carnivore	Raccoon dog	*Nyctereutes procyonoides*	Vorpommern-Rügen	5	positive	40	positive	-	negative	negative	negative
2024BVD04036	Carnivore	European badger	*Meles meles*	Vorpommern-Rügen	16	positive	5	positive	-	negative	negative	negative
2024BVD04037	Carnivore	Raccoon dog	*Nyctereutes procyonoides*	Vorpommern-Rügen	57	negative	-	-	-	negative	negative	negative
2024BVD04038	Carnivore	Raccoon dog	*Nyctereutes procyonoides*	Vorpommern-Rügen	38	positive	31	positive	-	negative	negative	negative
2024BVD08288	Carnivore	Raccoon dog	*Nyctereutes procyonoides*	Vorpommern-Rügen	90	negative	-	-	-	negative	negative	negative
2024BVD10141	Carnivore	Raccoon dog	*Nyctereutes procyonoides*	Vorpommern-Rügen	83	negative	-	-	-	negative	negative	negative
2024BVD10142	Carnivore	Red fox	*Vulpes vulpes*	Vorpommern-Rügen	not taken	not taken	not taken	not taken	-	negative	negative	negative
2024BVD10143	Carnivore	Raccoon dog	*Nyctereutes procyonoides*	Vorpommern-Rügen	86	negative	-	-	-	negative	negative	negative
2024BVD10144	Carnivore	Red fox	*Vulpes vulpes*	Vorpommern-Rügen	79	negative	-	-	-	negative	negative	negative
2024BVD10145	Carnivore	Red fox	*Vulpes vulpes*	Vorpommern-Rügen	58	negative	-	-	-	negative	negative	negative
2024BVD10146	Carnivore	Red fox	*Vulpes vulpes*	Vorpommern-Rügen	86	negative	-	-	-	negative	negative	negative
2024BVD10147	Carnivore	Red fox	*Vulpes vulpes*	Vorpommern-Rügen	110	negative	-	-	-	negative	negative	not taken
2024BVD10148	Carnivore	Red fox	*Vulpes vulpes*	Vorpommern-Rügen	87	negative	-	-	-	negative	negative	negative
2024BVD10149	Carnivore	Red fox	*Vulpes vulpes*	Vorpommern-Rügen	95	negative	-	-	-	negative	negative	negative
2024BVD10150	Carnivore	Red fox	*Vulpes vulpes*	Vorpommern-Rügen	80	negative	-	-	-	negative	negative	negative
2024BVD10151	Carnivore	Red fox	*Vulpes vulpes*	Vorpommern-Rügen	79	negative	-	-	-	negative	negative	negative
2024BVD10152	Carnivore	Red fox	*Vulpes vulpes*	Vorpommern-Rügen	93	negative	-	-	-	negative	negative	negative
2024BVD10153	Carnivore	Red fox	*Vulpes vulpes*	Vorpommern-Rügen	83	negative	-	-	-	negative	negative	negative
2024BVD10154	Carnivore	Red fox	*Vulpes vulpes*	Vorpommern-Rügen	88	negative	-	-	-	negative	negative	negative
2024BVD10155	Carnivore	Red fox	*Vulpes vulpes*	Vorpommern-Rügen	44	positive	77	negative	-	negative	negative	negative
2024BVD10156	Carnivore	Red fox	*Vulpes vulpes*	Vorpommern-Rügen	77	negative	-	-	-	negative	negative	negative
2024BVD10157	Carnivore	Red fox	*Vulpes vulpes*	Vorpommern-Rügen	84	negative	-	-	-	negative	negative	negative
2024BVD10158	Carnivore	Raccoon dog	*Nyctereutes procyonoides*	Vorpommern-Rügen	60	negative	-	-	-	negative	negative	negative
2024BVD10159	Carnivore	Red fox	*Vulpes vulpes*	Vorpommern-Rügen	25	positive	94	negative	<1:20	negative	negative	negative
2024BVD10160	Carnivore	Red fox	*Vulpes vulpes*	Vorpommern-Rügen	68	negative	-	-	-	negative	negative	negative
2024BVD10161	Carnivore	Red fox	*Vulpes vulpes*	Vorpommern-Rügen	45	undetermined	105	negative	-	negative	negative	negative

Sample ID	Group	Species	Scientific species name	Sample origin rural district	Result IAV-NP antibody ELISA in percent*	Assessment IAV-NP antibody ELISA	Result IAV-H5 antibody ELISA in percent†	Assessment IAV-H5 antibody ELISA	Titer serum neutralization assay	IAV RT-qPCR nasal swab	IAV RT-qPCR lung	IAV RT-qPCR brain
2024BVD10162	Carnivore	Raccoon dog	*Nyctereutes procyonoides*	Vorpommern-Rügen	87	negative	-	-	-	negative	negative	negative
2024BVD10163	Carnivore	Red fox	*Vulpes vulpes*	Vorpommern-Rügen	17	positive	81	negative	<1:20	negative	negative	negative
2024BVD10164	Carnivore	Red fox	*Vulpes vulpes*	Vorpommern-Rügen	28	positive	81	negative	<1:20	negative	negative	negative
2024BVD10165	Carnivore	Red fox	*Vulpes vulpes*	Vorpommern-Rügen	68	negative	-	-	-	negative	negative	negative
2024BVD12400	Carnivore	Red fox	*Vulpes vulpes*	Vorpommern-Rügen	29	positive	88	negative	-	negative	negative	negative
2024BVD12401	Carnivore	Red fox	*Vulpes vulpes*	Vorpommern-Rügen	41	positive	87	negative	<1:20	negative	negative	negative
2024BVD12402	Carnivore	Red fox	*Vulpes vulpes*	Vorpommern-Rügen	60	negative	-	-	-	negative	negative	negative
2024BVD12502	Carnivore	Red fox	*Vulpes vulpes*	Vorpommern-Rügen	47	undetermined	not available	not available	-	negative	negative	negative
2024BVD12503	Carnivore	Red fox	*Vulpes vulpes*	Vorpommern-Rügen	not taken	not taken	not taken	not taken	-	negative	negative	negative
2024BVD12504	Carnivore	Raccoon dog	*Nyctereutes procyonoides*	Vorpommern-Rügen	13	positive	5	positive	-	negative	negative	negative
2024BVD12505	Carnivore	Raccoon dog	*Nyctereutes procyonoides*	Vorpommern-Rügen	92	negative	-	-	-	negative	negative	negative
2024BVD12506	Carnivore	Raccoon dog	*Nyctereutes procyonoides*	Vorpommern-Rügen	78	negative	-	-	-	negative	not taken	negative
2024BVD12507	Carnivore	European badger	*Meles meles*	Vorpommern-Rügen	103	negative	-	-	-	negative	negative	not taken
2024BVD12508	Carnivore	Red fox	*Vulpes vulpes*	Vorpommern-Rügen	92	negative	-	-	-	negative	negative	negative
2024BVD12509	Carnivore	Red fox	*Vulpes vulpes*	Vorpommern-Rügen	98	negative	-	-	-	negative	negative	negative
2024BVD12510	Carnivore	Red fox	*Vulpes vulpes*	Vorpommern-Rügen	99	negative	-	-	-	negative	negative	negative
2024BVD12511	Carnivore	Red fox	*Vulpes vulpes*	Vorpommern-Rügen	85	negative	-	-	-	negative	negative	negative
2024BVD12512	Carnivore	Red fox	*Vulpes vulpes*	Vorpommern-Rügen	107	negative	-	-	-	negative	negative	negative
2024BVD12513	Carnivore	Red fox	*Vulpes vulpes*	Vorpommern-Rügen	92	negative	-	-	-	negative	negative	negative
2024BVD12514	Carnivore	Red fox	*Vulpes vulpes*	Vorpommern-Rügen	70	negative	-	-	-	negative	negative	negative
2024BVD12515	Carnivore	Red fox	*Vulpes vulpes*	Vorpommern-Rügen	94	negative	-	-	-	negative	negative	negative
2024BVD12516	Carnivore	Red fox	*Vulpes vulpes*	Vorpommern-Rügen	not available	not available	not available	not available	-	negative	negative	negative
2024BVD12517	Carnivore	Red fox	*Vulpes vulpes*	Vorpommern-Rügen	91	negative	-	-	-	negative	negative	negative
2024BVD12518	Carnivore	Red fox	*Vulpes vulpes*	Vorpommern-Rügen	not taken	not taken	not taken	not taken	-	negative	negative	negative
2024BVD12519	Carnivore	Raccoon dog	*Nyctereutes procyonoides*	Vorpommern-Rügen	86	negative	-	-	-	negative	negative	negative
2024BVD12520	Carnivore	Red fox	*Vulpes vulpes*	Vorpommern-Rügen	103	negative	-	-	-	negative	negative	negative
2024BVD12521	Carnivore	Red fox	*Vulpes vulpes*	Vorpommern-Rügen	72	negative	-	-	-	negative	negative	negative
2024BVD12522	Carnivore	Red fox	*Vulpes vulpes*	Vorpommern-Rügen	not taken	not taken	not taken	not taken	-	negative	not taken	not taken
2024BVD12523	Carnivore	Red fox	*Vulpes vulpes*	Vorpommern-Rügen	98	negative	-	-	-	negative	negative	negative
2024BVD12524	Carnivore	Red fox	*Vulpes vulpes*	Vorpommern-Rügen	89	negative	-	-	-	negative	negative	negative
2024BVD12525	Carnivore	Red fox	*Vulpes vulpes*	Vorpommern-Rügen	87	negative	-	-	-	negative	negative	negative
2024BVD12526	Carnivore	Raccoon dog	*Nyctereutes procyonoides*	Vorpommern-Rügen	not available	not available	not available	not available	-	negative	negative	negative
2024BVD12527	Carnivore	Raccoon dog	*Nyctereutes procyonoides*	Vorpommern-Rügen	96	negative	-	-	-	negative	negative	negative
2024BVD12528	Carnivore	Raccoon dog	*Nyctereutes procyonoides*	Vorpommern-Rügen	101	negative	-	-	-	negative	negative	negative

Sample ID	Group	Species	Scientific species name	Sample origin rural district	Result IAV-NP antibody ELISA in percent*	Assessment IAV-NP antibody ELISA	Result IAV-H5 antibody ELISA in percent†	Assessment IAV-H5 antibody ELISA	Titer serum neutralization assay	IAV RT-qPCR nasal swab	IAV RT-qPCR lung	IAV RT-qPCR brain
2024BVD12529	Carnivore	Raccoon dog	*Nyctereutes procyonoides*	Vorpommern-Rügen	96	negative	-	-	-	negative	negative	negative
2024BVD12530	Carnivore	Raccoon dog	*Nyctereutes procyonoides*	Vorpommern-Rügen	87	negative	-	-	-	negative	negative	negative
2024BVD12531	Carnivore	Raccoon dog	*Nyctereutes procyonoides*	Vorpommern-Rügen	82	negative	-	-	-	negative	negative	negative
2024BVD12532	Carnivore	Raccoon dog	*Nyctereutes procyonoides*	Vorpommern-Rügen	83	negative	-	-	-	negative	negative	negative
2024BVD12533	Carnivore	Red fox	*Vulpes vulpes*	Vorpommern-Rügen	81	negative	-	-	-	negative	negative	negative
2024BVD12534	Carnivore	Red fox	*Vulpes vulpes*	Vorpommern-Rügen	80	negative	-	-	-	negative	negative	negative
2024BVD12535	Carnivore	Red fox	*Vulpes vulpes*	Vorpommern-Rügen	70	negative	-	-	-	negative	negative	negative
2024BVD12536	Carnivore	Raccoon dog	*Nyctereutes procyonoides*	Vorpommern-Rügen	94	negative	-	-	-	negative	negative	negative
2024BVD12537	Carnivore	Raccoon dog	*Nyctereutes procyonoides*	Vorpommern-Rügen	95	negative	-	-	-	negative	negative	negative
2024BVD12538	Carnivore	Red fox	*Vulpes vulpes*	Vorpommern-Rügen	79	negative	-	-	-	negative	negative	negative
2024BVD12539	Carnivore	Red fox	*Vulpes vulpes*	Vorpommern-Rügen	95	negative	-	-	-	negative	negative	negative
2024BVD12540	Carnivore	Raccoon dog	*Nyctereutes procyonoides*	Vorpommern-Rügen	102	negative	-	-	-	negative	negative	negative
2024BVD12541	Carnivore	Raccoon dog	*Nyctereutes procyonoides*	Vorpommern-Rügen	91	negative	-	-	-	negative	negative	negative
2024BVD12542	Carnivore	Raccoon dog	*Nyctereutes procyonoides*	Vorpommern-Rügen	83	negative	-	-	-	negative	negative	negative
2024BVD12543	Carnivore	Red fox	*Vulpes vulpes*	Vorpommern-Rügen	11	positive	94	negative	-	negative	negative	negative
2024BVD12544	Carnivore	Red fox	*Vulpes vulpes*	Vorpommern-Rügen	2	positive	95	negative	-	negative	negative	negative
2024BVD12545	Carnivore	Red fox	*Vulpes vulpes*	Vorpommern-Rügen	52	negative	-	-	-	negative	negative	negative
2024BVD12546	Carnivore	Red fox	*Vulpes vulpes*	Vorpommern-Rügen	41	positive	114	negative	<1:20	negative	negative	negative
2024BVD12547	Carnivore	Red fox	*Vulpes vulpes*	Vorpommern-Rügen	66	negative	-	-	-	negative	negative	not taken
2024BVD12548	Carnivore	Raccoon dog	*Nyctereutes procyonoides*	Vorpommern-Rügen	73	negative	-	-	-	negative	negative	negative
2024BVD12549	Carnivore	Red fox	*Vulpes vulpes*	Vorpommern-Rügen	59	negative	-	-	-	negative	negative	negative
2024BVD12550	Carnivore	Red fox	*Vulpes vulpes*	Vorpommern-Rügen	83	negative	-	-	-	negative	negative	negative
2024BVD12551	Carnivore	Red fox	*Vulpes vulpes*	Vorpommern-Rügen	80	negative	-	-	-	negative	negative	negative
2024BVD12657	Carnivore	Red fox	*Vulpes vulpes*	Vorpommern-Rügen	95	negative	-	-	-	negative	negative	negative
2024BVD12658	Carnivore	Red fox	*Vulpes vulpes*	Vorpommern-Rügen	90	negative	-	-	-	negative	negative	negative
2024BVD12659	Carnivore	Red fox	*Vulpes vulpes*	Vorpommern-Rügen	97	negative	-	-	-	negative	negative	negative
2024BVD12660	Carnivore	Raccoon dog	*Nyctereutes procyonoides*	Vorpommern-Rügen	91	negative	-	-	-	negative	negative	not taken
2024BVD12661	Carnivore	Red fox	*Vulpes vulpes*	Vorpommern-Rügen	97	negative	-	-	-	negative	negative	negative
2024BVD12662	Carnivore	Red fox	*Vulpes vulpes*	Vorpommern-Rügen	94	negative	-	-	-	negative	negative	negative
2024BVD12663	Carnivore	European pine marten	*Martes martes*	Vorpommern-Rügen	91	negative	-	-	-	negative	negative	negative
2024BVD12664	Carnivore	Red fox	*Vulpes vulpes*	Vorpommern-Rügen	85	negative	-	-	-	negative	negative	negative
2024BVD12665	Carnivore	Red fox	*Vulpes vulpes*	Vorpommern-Rügen	22	positive	6	positive	-	negative	negative	negative

Sample ID	Group	Species	Scientific species name	Sample origin rural district	Result IAV-NP antibody ELISA in percent*	Assessment IAV-NP antibody ELISA	Result IAV-H5 antibody ELISA in percent†	Assessment IAV-H5 antibody ELISA	Titer serum neutralization assay	IAV RT-qPCR nasal swab	IAV RT-qPCR lung	IAV RT-qPCR brain
2024BVD12667	Carnivore	Raccoon dog	*Nyctereutes procyonoides*	Vorpommern-Rügen	not taken	not taken	not taken	not taken	-	negative	negative	negative
2024BVD12668	Carnivore	Red fox	*Vulpes vulpes*	Vorpommern-Rügen	75	negative	-	-	-	negative	negative	negative
2024BVD12669	Carnivore	Red fox	*Vulpes vulpes*	Vorpommern-Rügen	66	negative	-	-	-	negative	negative	negative
2024BVD12670	Carnivore	Raccoon dog	*Nyctereutes procyonoides*	Vorpommern-Rügen	64	negative	-	-	-	negative	negative	negative
2024BVD12671	Carnivore	European badger	*Meles meles*	Vorpommern-Rügen	75	negative	-	-	-	negative	negative	negative
2024BVD12672	Carnivore	Raccoon dog	*Nyctereutes procyonoides*	Vorpommern-Rügen	63	negative	-	-	-	negative	negative	negative
2024BVD12673	Carnivore	Red fox	*Vulpes vulpes*	Vorpommern-Rügen	91	negative	-	-	-	negative	negative	negative
2024BVD12674	Carnivore	Raccoon dog	*Nyctereutes procyonoides*	Vorpommern-Rügen	87	negative	-	-	-	negative	negative	negative
2024BVD12675	Carnivore	Raccoon dog	*Nyctereutes procyonoides*	Vorpommern-Rügen	97	negative	-	-	-	negative	negative	negative
2024BVD12676	Carnivore	Red fox	*Vulpes vulpes*	Vorpommern-Rügen	94	negative	-	-	-	negative	negative	negative
2024BVD12677	Carnivore	Red fox	*Vulpes vulpes*	Vorpommern-Rügen	83	negative	-	-	-	negative	negative	negative
2024BVD12678	Carnivore	Raccoon dog	*Nyctereutes procyonoides*	Vorpommern-Rügen	90	negative	-	-	-	negative	negative	negative
2024BVD12679	Carnivore	Red fox	*Vulpes vulpes*	Vorpommern-Rügen	88	negative	-	-	-	negative	negative	negative
2024BVD12680	Carnivore	Red fox	*Vulpes vulpes*	Vorpommern-Rügen	85	negative	-	-	-	negative	negative	negative
2024BVD12681	Carnivore	Red fox	*Vulpes vulpes*	Vorpommern-Rügen	47	undetermined	not available	not available	-	negative	negative	negative
2024BVD12682	Carnivore	Raccoon	*Procyon lotor*	Vorpommern-Rügen	95	negative	-	-	-	negative	negative	negative
2024BVD12683	Carnivore	Raccoon dog	*Nyctereutes procyonoides*	Vorpommern-Rügen	not taken	not taken	not taken	not taken	-	negative	negative	negative
2024BVD12684	Carnivore	Red fox	*Vulpes vulpes*	Vorpommern-Rügen	64	negative	-	-	-	negative	negative	negative
2024BVD12685	Carnivore	Raccoon dog	*Nyctereutes procyonoides*	Vorpommern-Rügen	89	negative	-	-	-	negative	negative	negative
2024BVD12686	Carnivore	Raccoon dog	*Nyctereutes procyonoides*	Vorpommern-Rügen	69	negative	-	-	-	negative	negative	negative
2024BVD12687	Carnivore	Raccoon	*Procyon lotor*	Vorpommern-Rügen	85	negative	-	-	-	negative	negative	negative
2024BVD12688	Carnivore	Raccoon dog	*Nyctereutes procyonoides*	Vorpommern-Rügen	83	negative	-	-	-	negative	negative	negative
2024BVD12689	Carnivore	Red fox	*Vulpes vulpes*	Vorpommern-Rügen	91	negative	-	-	-	negative	negative	negative
2024BVD12690	Carnivore	Red fox	*Vulpes vulpes*	Vorpommern-Rügen	95	negative	-	-	-	negative	negative	negative
2024BVD12691	Carnivore	Red fox	*Vulpes vulpes*	Vorpommern-Rügen	96	negative	-	-	-	negative	negative	negative
2024BVD12692	Carnivore	Red fox	*Vulpes vulpes*	Vorpommern-Rügen	99	negative	-	-	-	negative	negative	negative
2024BVD12693	Carnivore	Raccoon	*Procyon lotor*	Vorpommern-Rügen	101	negative	-	-	-	negative	negative	negative
2024BVD12694	Carnivore	Raccoon	*Procyon lotor*	Vorpommern-Rügen	92	negative	-	-	-	negative	negative	negative
2024BVD12695	Carnivore	Raccoon dog	*Nyctereutes procyonoides*	Vorpommern-Rügen	87	negative	-	-	-	negative	negative	negative
2024BVD12696	Carnivore	Red fox	*Vulpes vulpes*	Vorpommern-Rügen	91	negative	-	-	-	negative	negative	negative
2024BVD12697	Carnivore	Raccoon dog	*Nyctereutes procyonoides*	Vorpommern-Rügen	not taken	not taken	not taken	not taken	-	negative	negative	negative

Sample ID	Group	Species	Scientific species name	Sample origin rural district	Result IAV-NP antibody ELISA in percent*	Assessment IAV-NP antibody ELISA	Result IAV-H5 antibody ELISA in percent†	Assessment IAV-H5 antibody ELISA	Titer serum neutralization assay	IAV RT-qPCR nasal swab	IAV RT-qPCR lung	IAV RT-qPCR brain
2024BVD12698	Carnivore	Raccoon dog	*Nyctereutes procyonoides*	Vorpommern-Rügen	90	negative	-	-	-	negative	negative	negative
2024BVD12699	Carnivore	Red fox	*Vulpes vulpes*	Vorpommern-Rügen	95	negative	-	-	-	negative	negative	negative
2024BVD12700	Carnivore	Raccoon dog	*Nyctereutes procyonoides*	Vorpommern-Rügen	87	negative	-	-	-	negative	negative	negative
2024BVD12701	Carnivore	Raccoon dog	*Nyctereutes procyonoides*	Vorpommern-Rügen	23	positive	17	positive	-	negative	negative	negative
2024BVD15064	Carnivore	Raccoon dog	*Nyctereutes procyonoides*	Vorpommern-Rügen	96	negative	-	-	-	negative	negative	negative
2024BVD15065	Carnivore	Red fox	*Vulpes vulpes*	Vorpommern-Rügen	82	negative	-	-	-	negative	negative	negative
2024BVD15066	Carnivore	Raccoon dog	*Nyctereutes procyonoides*	Vorpommern-Rügen	96	negative	-	-	-	negative	negative	negative
2024BVD15067	Carnivore	Raccoon	*Procyon lotor*	Vorpommern-Rügen	98	negative	-	-	-	negative	negative	negative
2024BVD15068	Carnivore	Raccoon dog	*Nyctereutes procyonoides*	Vorpommern-Rügen	10	positive	5	positive	-	negative	negative	negative
2024BVD15069	Carnivore	Raccoon	*Procyon lotor*	Vorpommern-Rügen	89	negative	-	-	-	negative	negative	negative
2024BVD15070	Carnivore	Raccoon dog	*Nyctereutes procyonoides*	Vorpommern-Rügen	67	negative	-	-	-	negative	negative	negative
2024BVD15071	Carnivore	Red fox	*Vulpes vulpes*	Vorpommern-Rügen	97	negative	-	-	-	negative	negative	negative
2024BVD15072	Carnivore	Raccoon dog	*Nyctereutes procyonoides*	Vorpommern-Rügen	91	negative	-	-	-	negative	negative	negative
2024BVD15073	Carnivore	Red fox	*Vulpes vulpes*	Vorpommern-Rügen	57	negative	-	-	-	negative	negative	negative
2024BVD15074	Carnivore	Raccoon dog	*Nyctereutes procyonoides*	Vorpommern-Rügen	110	negative	-	-	-	negative	negative	negative
2024BVD15075	Carnivore	Red fox	*Vulpes vulpes*	Vorpommern-Rügen	100	negative	-	-	-	negative	negative	negative
2024BVD15076	Carnivore	Raccoon dog	*Nyctereutes procyonoides*	Vorpommern-Rügen	103	negative	-	-	-	negative	negative	negative
2024BVD15077	Carnivore	Raccoon dog	*Nyctereutes procyonoides*	Vorpommern-Rügen	98	negative	-	-	-	negative	negative	negative
2024BVD15078	Carnivore	Raccoon	*Procyon lotor*	Vorpommern-Rügen	92	negative	-	-	-	negative	negative	not taken
2024BVD15079	Carnivore	Red fox	*Vulpes vulpes*	Vorpommern-Rügen	99	negative	-	-	-	negative	negative	negative
2024BVD15080	Carnivore	Raccoon	*Procyon lotor*	Vorpommern-Rügen	11	positive	4	positive	-	negative	negative	negative
2024BVD15081	Carnivore	Raccoon dog	*Nyctereutes procyonoides*	Vorpommern-Rügen	105	negative	-	-	-	negative	negative	negative
2024BVD15082	Carnivore	Red fox	*Vulpes vulpes*	Vorpommern-Rügen	103	negative	-	-	-	negative	negative	negative
2024BVD15083	Carnivore	Raccoon dog	*Nyctereutes procyonoides*	Vorpommern-Rügen	103	negative	-	-	-	negative	negative	negative
2024BVD15084	Carnivore	Raccoon dog	*Nyctereutes procyonoides*	Vorpommern-Rügen	106	negative	-	-	-	negative	negative	negative
2024BVD15085	Carnivore	Red fox	*Vulpes vulpes*	Vorpommern-Rügen	75	negative	-	-	-	negative	negative	negative
2024BVD15086	Carnivore	Red fox	*Vulpes vulpes*	Vorpommern-Rügen	84	negative	-	-	-	negative	negative	negative
2024BVD15087	Carnivore	Raccoon dog	*Nyctereutes procyonoides*	Vorpommern-Rügen	82	negative	-	-	-	negative	negative	negative
2024BVD15088	Carnivore	Raccoon dog	*Nyctereutes procyonoides*	Vorpommern-Rügen	95	negative	-	-	-	negative	negative	negative

Sample ID	Group	Species	Scientific species name	Sample origin rural district	Result IAV-NP antibody ELISA in percent*	Assessment IAV-NP antibody ELISA	Result IAV-H5 antibody ELISA in percent†	Assessment IAV-H5 antibody ELISA	Titer serum neutralization assay	IAV RT-qPCR nasal swab	IAV RT-qPCR lung	IAV RT-qPCR brain
2024BVD15089	Carnivore	Raccoon dog	*Nyctereutes procyonoides*	Vorpommern-Rügen	4	positive	5	positive	-	negative	negative	negative
2024BVD15090	Carnivore	Raccoon dog	*Nyctereutes procyonoides*	Vorpommern-Rügen	101	negative	-	-	-	negative	negative	negative
2024BVD15091	Carnivore	Raccoon dog	*Nyctereutes procyonoides*	Vorpommern-Rügen	86	negative	-	-	-	negative	negative	negative
2024BVD15092	Carnivore	Raccoon dog	*Nyctereutes procyonoides*	Vorpommern-Rügen	100	negative	-	-	-	negative	negative	negative
2024BVD15093	Carnivore	Raccoon dog	*Nyctereutes procyonoides*	Vorpommern-Rügen	95	negative	-	-	-	negative	negative	negative
2024BVD15094	Carnivore	Red fox	*Vulpes vulpes*	Vorpommern-Rügen	90	negative	-	-	-	negative	negative	negative
2024BVD15095	Carnivore	Raccoon dog	*Nyctereutes procyonoides*	Vorpommern-Rügen	83	negative	-	-	-	negative	negative	negative
2024BVD15096	Carnivore	Raccoon dog	*Nyctereutes procyonoides*	Vorpommern-Rügen	10	positive	9	positive	-	negative	negative	negative
2024BVD15097	Carnivore	Raccoon dog	*Nyctereutes procyonoides*	Vorpommern-Rügen	91	negative	-	-	-	negative	negative	negative
2024BVD15098	Carnivore	Red fox	*Vulpes vulpes*	Vorpommern-Rügen	97	negative	-	-	-	negative	negative	negative
2024BVD15099	Carnivore	Raccoon dog	*Nyctereutes procyonoides*	Vorpommern-Rügen	89	negative	-	-	-	negative	negative	negative
2024BVD15100	Carnivore	Red fox	*Vulpes vulpes*	Vorpommern-Rügen	102	negative	-	-	-	negative	negative	negative
2024BVD15101	Carnivore	Red fox	*Vulpes vulpes*	Vorpommern-Rügen	5	positive	6	positive	-	negative	negative	negative
2024BVD15102	Carnivore	Red fox	*Vulpes vulpes*	Vorpommern-Rügen	108	negative	-	-	-	negative	negative	negative
2024BVD15103	Carnivore	Red fox	*Vulpes vulpes*	Vorpommern-Rügen	103	negative	-	-	-	negative	negative	negative
2024BVD15104	Carnivore	Raccoon dog	*Nyctereutes procyonoides*	Vorpommern-Rügen	96	negative	-	-	-	negative	negative	negative
2024BVD15105	Carnivore	Raccoon	*Procyon lotor*	Vorpommern-Rügen	106	negative	-	-	-	negative	negative	negative
2024BVD15106	Carnivore	Raccoon	*Procyon lotor*	Vorpommern-Rügen	36	positive	93	negative	<1:20	negative	negative	negative
2024BVD15107	Carnivore	Raccoon	*Procyon lotor*	Vorpommern-Rügen	80	negative	-	-	-	negative	negative	negative
2024BVD15108	Carnivore	Red fox	*Vulpes vulpes*	Vorpommern-Rügen	21	positive	61	negative	<1:20	negative	negative	negative
2024BVD16173	Carnivore	Raccoon	*Procyon lotor*	Vorpommern-Rügen	30	positive	99	negative	-	negative	negative	negative
2024BVD16174	Carnivore	Red fox	*Vulpes vulpes*	Vorpommern-Rügen	4	positive	80	negative	-	negative	negative	negative
2024BVD16175	Carnivore	Raccoon dog	*Nyctereutes procyonoides*	Vorpommern-Rügen	87	negative	-	-	-	negative	negative	negative
2024BVD16176	Carnivore	Red fox	*Vulpes vulpes*	Vorpommern-Rügen	52	negative	-	-	-	negative	negative	negative
2024BVD16177	Carnivore	Red fox	*Vulpes vulpes*	Vorpommern-Rügen	16	positive	32	positive	-	negative	negative	negative
2024BVD16178	Carnivore	Raccoon dog	*Nyctereutes procyonoides*	Vorpommern-Rügen	94	negative	-	-	-	negative	not taken	negative
2024BVD16179	Carnivore	Raccoon dog	*Nyctereutes procyonoides*	Vorpommern-Rügen	96	negative	-	-	-	negative	negative	negative
2024BVD16180	Carnivore	Raccoon dog	*Nyctereutes procyonoides*	Vorpommern-Rügen	93	negative	-	-	-	negative	negative	negative
2024BVD16181	Carnivore	Raccoon	*Procyon lotor*	Vorpommern-Rügen	12	positive	5	positive	-	negative	negative	negative
2024BVD16182	Carnivore	Raccoon dog	*Nyctereutes procyonoides*	Vorpommern-Rügen	86	negative	-	-	-	negative	negative	negative

Sample ID	Group	Species	Scientific species name	Sample origin rural district	Result IAV-NP antibody ELISA in percent*	Assessment IAV-NP antibody ELISA	Result IAV-H5 antibody ELISA in percent†	Assessment IAV-H5 antibody ELISA	Titer serum neutralization assay	IAV RT-qPCR nasal swab	IAV RT-qPCR lung	IAV RT-qPCR brain
2024BVD16183	Carnivore	Raccoon dog	*Nyctereutes procyonoides*	Vorpommern-Rügen	85	negative	-	-	-	negative	negative	negative
2024BVD16184	Carnivore	Raccoon dog	*Nyctereutes procyonoides*	Vorpommern-Rügen	92	negative	-	-	-	negative	negative	negative
2024BVD16185	Carnivore	Raccoon dog	*Nyctereutes procyonoides*	Vorpommern-Rügen	90	negative	-	-	-	negative	negative	negative
2024BVD16186	Carnivore	Red fox	*Vulpes vulpes*	Vorpommern-Rügen	79	negative	-	-	-	negative	negative	negative
2024BVD16187	Carnivore	European badger	*Meles meles*	Vorpommern-Rügen	83	negative	-	-	-	negative	negative	negative
2024BVD16188	Carnivore	Raccoon dog	*Nyctereutes procyonoides*	Vorpommern-Rügen	88	negative	-	-	-	negative	negative	negative
2024BVD16189	Carnivore	Raccoon	*Procyon lotor*	Vorpommern-Rügen	4	positive	4	positive	≥1:160	negative	negative	negative
2024BVD16190	Carnivore	Red fox	*Vulpes vulpes*	Vorpommern-Rügen	31	positive	5	positive	-	negative	negative	negative
2024BVD16191	Carnivore	Raccoon dog	*Nyctereutes procyonoides*	Vorpommern-Rügen	102	negative	-	-	-	negative	negative	negative
2024BVD16192	Carnivore	Raccoon	*Procyon lotor*	Vorpommern-Rügen	89	negative	-	-	-	negative	negative	negative
2024BVD16193	Carnivore	Raccoon dog	*Nyctereutes procyonoides*	Vorpommern-Rügen	83	negative	-	-	-	negative	negative	negative
2024BVD16194	Carnivore	Raccoon dog	*Nyctereutes procyonoides*	Vorpommern-Rügen	83	negative	-	-	-	negative	negative	negative
2024BVD16195	Carnivore	Red fox	*Vulpes vulpes*	Vorpommern-Rügen	44	positive	5	positive	-	negative	negative	negative
2024BVD16196	Carnivore	European badger	*Meles meles*	Vorpommern-Rügen	85	negative	-	-	-	negative	negative	negative
2024BVD16197	Carnivore	Red fox	*Vulpes vulpes*	Vorpommern-Rügen	77	negative	-	-	-	negative	negative	negative
2024BVD16198	Carnivore	Red fox	*Vulpes vulpes*	Vorpommern-Rügen	79	negative	-	-	-	negative	negative	negative
2024BVD16199	Carnivore	Raccoon dog	*Nyctereutes procyonoides*	Vorpommern-Rügen	5	positive	5	positive	-	negative	negative	negative
2024BVD16200	Carnivore	Raccoon dog	*Nyctereutes procyonoides*	Vorpommern-Rügen	89	negative	-	-	-	negative	negative	negative
2024BVD16201	Carnivore	Raccoon dog	*Nyctereutes procyonoides*	Vorpommern-Rügen	103	negative	-	-	-	negative	negative	negative
2024BVD16202	Carnivore	Raccoon	*Procyon lotor*	Vorpommern-Rügen	13	positive	50 (50.02)	undetermined	-	negative	negative	negative
2024BVD16203	Carnivore	Red fox	*Vulpes vulpes*	Vorpommern-Rügen	104	negative	-	-	-	negative	negative	negative
2024BVD16204	Carnivore	Red fox	*Vulpes vulpes*	Vorpommern-Rügen	99	negative	-	-	-	negative	negative	negative
2024BVD16205	Carnivore	Raccoon dog	*Nyctereutes procyonoides*	Vorpommern-Rügen	97	negative	-	-	-	negative	negative	negative
2024BVD16206	Carnivore	Raccoon dog	*Nyctereutes procyonoides*	Vorpommern-Rügen	91	negative	-	-	-	negative	negative	negative
2024BVD16207	Carnivore	Red fox	*Vulpes vulpes*	Vorpommern-Rügen	39	positive	17	positive	-	negative	negative	negative
2024BVD16208	Carnivore	Red fox	*Vulpes vulpes*	Vorpommern-Rügen	81	negative	-	-	-	negative	not taken	negative
2024BVD16209	Carnivore	Raccoon	*Procyon lotor*	Vorpommern-Rügen	111	negative	-	-	-	negative	negative	negative
2024BVD16210	Carnivore	Raccoon dog	*Nyctereutes procyonoides*	Vorpommern-Rügen	98	negative	-	-	-	negative	negative	negative
2024BVD16211	Carnivore	Raccoon dog	*Nyctereutes procyonoides*	Vorpommern-Rügen	93	negative	-	-	-	negative	negative	negative

Sample ID	Group	Species	Scientific species name	Sample origin rural district	Result IAV-NP antibody ELISA in percent*	Assessment IAV-NP antibody ELISA	Result IAV-H5 antibody ELISA in percent†	Assessment IAV-H5 antibody ELISA	Titer serum neutralization assay	IAV RT-qPCR nasal swab	IAV RT-qPCR lung	IAV RT-qPCR brain
2024BVD16212	Carnivore	Red fox	*Vulpes vulpes*	Vorpommern-Rügen	88	negative	-	-	-	negative	negative	negative
2024BVD16213	Carnivore	Raccoon dog	*Nyctereutes procyonoides*	Vorpommern-Rügen	96	negative	-	-	-	negative	negative	negative
2024BVD16214	Carnivore	Raccoon dog	*Nyctereutes procyonoides*	Vorpommern-Rügen	58	negative	-	-	-	negative	negative	negative
2024BVD16215	Carnivore	Raccoon dog	*Nyctereutes procyonoides*	Vorpommern-Rügen	81	negative	-	-	-	negative	negative	negative
2024BVD16216	Carnivore	Raccoon dog	*Nyctereutes procyonoides*	Vorpommern-Rügen	10	positive	12	positive	-	negative	negative	not taken
2024BVD16217	Carnivore	Raccoon dog	*Nyctereutes procyonoides*	Vorpommern-Rügen	102	negative	-	-	-	negative	negative	negative
2024BVD16218	Carnivore	Red fox	*Vulpes vulpes*	Vorpommern-Rügen	84	negative	-	-	-	negative	negative	negative
2024BVD16219	Carnivore	Red fox	*Vulpes vulpes*	Vorpommern-Rügen	97	negative	-	-	-	negative	negative	negative
2024BVD16220	Carnivore	Raccoon	*Procyon lotor*	Vorpommern-Rügen	4	positive	5	positive	-	negative	negative	negative
2024BVD16221	Carnivore	Red fox	*Vulpes vulpes*	Vorpommern-Rügen	98	negative	-	-	-	negative	negative	negative
2024BVD16222	Carnivore	Raccoon dog	*Nyctereutes procyonoides*	Vorpommern-Rügen	27	positive	76	negative	-	negative	negative	negative
2024BVD16376	Carnivore	Red fox	*Vulpes vulpes*	Vorpommern-Rügen	58	negative	-	-	-	negative	negative	negative
2024BVD16377	Carnivore	Raccoon	*Procyon lotor*	Vorpommern-Rügen	90	negative	-	-	-	negative	negative	negative
2024BVD16378	Carnivore	Red fox	*Vulpes vulpes*	Vorpommern-Rügen	60	negative	-	-	-	negative	negative	negative
2024BVD16379	Carnivore	Red fox	*Vulpes vulpes*	Vorpommern-Rügen	73	negative	-	-	-	negative	negative	negative
2024BVD16380	Carnivore	Raccoon dog	*Nyctereutes procyonoides*	Vorpommern-Rügen	101	negative	-	-	-	negative	negative	negative
2024BVD16381	Carnivore	Raccoon dog	*Nyctereutes procyonoides*	Vorpommern-Rügen	91	negative	-	-	-	negative	negative	negative
2024BVD16382	Carnivore	Red fox	*Vulpes vulpes*	Vorpommern-Rügen	85	negative	-	-	-	negative	negative	negative
2024BVD16383	Carnivore	Raccoon dog	*Nyctereutes procyonoides*	Vorpommern-Rügen	93	negative	-	-	-	negative	negative	not taken
2024BVD16384	Carnivore	Raccoon dog	*Nyctereutes procyonoides*	Vorpommern-Rügen	not taken	not taken	not taken	not taken	-	negative	negative	negative
2024BVD16385	Carnivore	Raccoon dog	*Nyctereutes procyonoides*	Vorpommern-Rügen	107	negative	-	-	-	negative	negative	negative
2024BVD16386	Carnivore	Red fox	*Vulpes vulpes*	Vorpommern-Rügen	52	negative	-	-	-	negative	negative	negative
2024BVD16387	Carnivore	Raccoon dog	*Nyctereutes procyonoides*	Vorpommern-Rügen	not taken	not taken	not taken	not taken	-	negative	negative	negative
2024BVD16388	Carnivore	Raccoon dog	*Nyctereutes procyonoides*	Vorpommern-Rügen	6	positive	5	positive	-	negative	negative	negative
2024BVD16389	Carnivore	Raccoon dog	*Nyctereutes procyonoides*	Vorpommern-Rügen	101	negative	-	-	-	negative	negative	negative
2024BVD16390	Carnivore	Raccoon dog	*Nyctereutes procyonoides*	Vorpommern-Rügen	62	negative	-	-	-	negative	negative	negative
2024BVD16391	Carnivore	Red fox	*Vulpes vulpes*	Vorpommern-Rügen	13	positive	5	positive	-	negative	negative	negative
2024BVD16392	Carnivore	Raccoon	*Procyon lotor*	Vorpommern-Rügen	105	negative	-	-	-	negative	negative	negative
2024BVD16393	Carnivore	Red fox	*Vulpes vulpes*	Vorpommern-Rügen	69	negative	-	-	-	negative	negative	negative

Sample ID	Group	Species	Scientific species name	Sample origin rural district	Result IAV-NP antibody ELISA in percent*	Assessment IAV-NP antibody ELISA	Result IAV-H5 antibody ELISA in percent†	Assessment IAV-H5 antibody ELISA	Titer serum neutralization assay	IAV RT-qPCR nasal swab	IAV RT-qPCR lung	IAV RT-qPCR brain
2024BVD16394	Carnivore	Raccoon dog	*Nyctereutes procyonoides*	Vorpommern-Rügen	107	negative	-	-	-	negative	negative	negative
2024BVD16395	Carnivore	Red fox	*Vulpes vulpes*	Vorpommern-Rügen	86	negative	-	-	-	negative	negative	negative
2024BVD16396	Carnivore	Raccoon dog	*Nyctereutes procyonoides*	Vorpommern-Rügen	7	positive	6	positive	-	negative	negative	negative
2024BVD16397	Carnivore	Raccoon	*Procyon lotor*	Vorpommern-Rügen	not taken	not taken	not taken	not taken	-	negative	negative	negative
2024BVD16398	Carnivore	Red fox	*Vulpes vulpes*	Vorpommern-Rügen	82	negative	-	-	-	negative	negative	negative
2024BVD16399	Carnivore	Raccoon dog	*Nyctereutes procyonoides*	Vorpommern-Rügen	93	negative	-	-	-	negative	negative	negative
2024BVD16400	Carnivore	Red fox	*Vulpes vulpes*	Vorpommern-Rügen	100	negative	-	-	-	negative	negative	negative
2024BVD16401	Carnivore	Raccoon dog	*Nyctereutes procyonoides*	Vorpommern-Rügen	99	negative	-	-	-	negative	negative	negative
2024BVD16402	Carnivore	Red fox	*Vulpes vulpes*	Vorpommern-Rügen	77	negative	-	-	-	negative	negative	negative
2024BVD16403	Carnivore	Red fox	*Vulpes vulpes*	Vorpommern-Rügen	79	negative	-	-	-	negative	negative	negative
2024BVD16404	Carnivore	Raccoon dog	*Nyctereutes procyonoides*	Vorpommern-Rügen	81	negative	-	-	-	negative	negative	negative
2024BVD16405	Carnivore	Red fox	*Vulpes vulpes*	Vorpommern-Rügen	95	negative	-	-	-	negative	negative	negative
2024BVD16406	Carnivore	Raccoon dog	*Nyctereutes procyonoides*	Vorpommern-Rügen	94	negative	-	-	-	negative	negative	negative
2024BVD16407	Carnivore	Red fox	*Vulpes vulpes*	Vorpommern-Rügen	105	negative	-	-	-	negative	negative	negative
2024BVD16408	Carnivore	Raccoon dog	*Nyctereutes procyonoides*	Vorpommern-Rügen	93	negative	-	-	-	negative	negative	negative
2024BVD16409	Carnivore	Red fox	*Vulpes vulpes*	Vorpommern-Rügen	104	negative	-	-	-	negative	negative	negative
2024BVD16410	Carnivore	Red fox	*Vulpes vulpes*	Vorpommern-Rügen	106	negative	-	-	-	negative	negative	negative
2024BVD16411	Carnivore	Raccoon dog	*Nyctereutes procyonoides*	Vorpommern-Rügen	not taken	not taken	not taken	not taken	-	negative	negative	negative
2024BVD16412	Carnivore	Raccoon	*Procyon lotor*	Vorpommern-Rügen	107	negative	-	-	-	negative	negative	negative
2024BVD16413	Carnivore	Raccoon	*Procyon lotor*	Vorpommern-Rügen	109	negative	-	-	-	negative	negative	negative
2024BVD16414	Carnivore	Raccoon	*Procyon lotor*	Vorpommern-Rügen	15	positive	14	positive	-	negative	negative	negative
2024BVD16415	Carnivore	Red fox	*Vulpes vulpes*	Vorpommern-Rügen	103	negative	-	-	-	negative	negative	negative
2024BVD16416	Carnivore	Raccoon dog	*Nyctereutes procyonoides*	Vorpommern-Rügen	42	positive	6	positive	-	negative	negative	negative
2024BVD16417	Carnivore	Raccoon dog	*Nyctereutes procyonoides*	Vorpommern-Rügen	84	negative	-	-	-	negative	negative	negative
2024BVD16418	Carnivore	Raccoon dog	*Nyctereutes procyonoides*	Vorpommern-Rügen	38	positive	6	positive	-	negative	negative	negative
2024BVD16419	Carnivore	Raccoon dog	*Nyctereutes procyonoides*	Vorpommern-Rügen	100	negative	-	-	-	negative	negative	negative
2024BVD16420	Carnivore	Red fox	*Vulpes vulpes*	Vorpommern-Rügen	not taken	not taken	not taken	not taken	-	negative	negative	negative
2024BVD16421	Carnivore	Raccoon dog	*Nyctereutes procyonoides*	Vorpommern-Rügen	8	positive	5	positive	-	negative	negative	negative
2024BVD16422	Carnivore	Raccoon dog	*Nyctereutes procyonoides*	Vorpommern-Rügen	8	positive	5	positive	-	negative	negative	negative
2024BVD16423	Carnivore	Raccoon dog	*Nyctereutes procyonoides*	Vorpommern-Rügen	89	negative	-	-	-	negative	negative	negative

Sample ID	Group	Species	Scientific species name	Sample origin rural district	Result IAV-NP antibody ELISA in percent*	Assessment IAV-NP antibody ELISA	Result IAV-H5 antibody ELISA in percent†	Assessment IAV-H5 antibody ELISA	Titer serum neutralization assay	IAV RT-qPCR nasal swab	IAV RT-qPCR lung	IAV RT-qPCR brain
2024BVD16424	Carnivore	Raccoon dog	*Nyctereutes procyonoides*	Vorpommern-Rügen	100	negative	-	-	-	negative	negative	negative
2024BVD16425	Carnivore	Red fox	*Vulpes vulpes*	Vorpommern-Rügen	105	negative	-	-	-	negative	negative	negative
2024BVD16426	Carnivore	Red fox	*Vulpes vulpes*	Vorpommern-Rügen	not taken	not taken	not taken	not taken	-	negative	negative	negative
2024BVD16427	Carnivore	Raccoon dog	*Nyctereutes procyonoides*	Vorpommern-Rügen	16	positive	7	positive	-	negative	negative	negative
2024BVD16428	Carnivore	Red fox	*Vulpes vulpes*	Vorpommern-Rügen	102	negative	-	-	-	negative	negative	negative
2024BVD16429	Carnivore	Red fox	*Vulpes vulpes*	Vorpommern-Rügen	91	negative	-	-	-	negative	negative	negative
2024BVD16430	Carnivore	Raccoon dog	*Nyctereutes procyonoides*	Vorpommern-Rügen	97	negative	-	-	-	negative	negative	negative
2024BVD16431	Carnivore	Raccoon dog	*Nyctereutes procyonoides*	Vorpommern-Rügen	73	negative	-	-	-	negative	negative	negative
2024BVD16432	Carnivore	Raccoon	*Procyon lotor*	Vorpommern-Rügen	98	negative	-	-	-	negative	negative	negative
2024BVD16433	Carnivore	Raccoon	*Procyon lotor*	Vorpommern-Rügen	103	negative	-	-	-	negative	negative	negative
2024BVD16434	Carnivore	Raccoon	*Procyon lotor*	Vorpommern-Rügen	105	negative	-	-	-	negative	negative	negative
2024BVD16435	Carnivore	Red fox	*Vulpes vulpes*	Vorpommern-Rügen	23	positive	22	positive	-	negative	negative	negative
2024BVD16436	Carnivore	Raccoon	*Procyon lotor*	Vorpommern-Rügen	109	negative	-	-	-	negative	negative	negative
2024BVD16437	Carnivore	Red fox	*Vulpes vulpes*	Vorpommern-Rügen	104	negative	-	-	-	negative	negative	negative
2024BVD16438	Carnivore	Raccoon	*Procyon lotor*	Vorpommern-Rügen	105	negative	-	-	-	not taken	negative	not taken
2024BVD16439	Carnivore	Raccoon dog	*Nyctereutes procyonoides*	Vorpommern-Rügen	102	negative	-	-	-	negative	negative	negative
2024BVD16440	Carnivore	Raccoon	*Procyon lotor*	Vorpommern-Rügen	4	positive	4	positive	-	negative	negative	negative
2024BVD16441	Carnivore	Raccoon dog	*Nyctereutes procyonoides*	Vorpommern-Rügen	96	negative	-	-	-	negative	negative	negative
2024BVD16981	Carnivore	Raccoon	*Procyon lotor*	Vorpommern-Rügen	103	negative	-	-	-	negative	negative	negative
2024BVD16982	Carnivore	Red fox	*Vulpes vulpes*	Vorpommern-Rügen	79	negative	-	-	-	negative	negative	negative
2024BVD16983	Carnivore	Red fox	*Vulpes vulpes*	Vorpommern-Rügen	103	negative	-	-	-	negative	negative	negative
2024BVD16984	Carnivore	Raccoon dog	*Nyctereutes procyonoides*	Vorpommern-Rügen	106	negative	-	-	-	negative	negative	negative
2024BVD16985	Carnivore	Red fox	*Vulpes vulpes*	Vorpommern-Rügen	107	negative	-	-	-	negative	negative	negative
2024BVD16986	Carnivore	Red fox	*Vulpes vulpes*	Vorpommern-Rügen	96	negative	-	-	-	negative	negative	negative
2024BVD16987	Carnivore	Red fox	*Vulpes vulpes*	Vorpommern-Rügen	89	negative	-	-	-	negative	negative	negative
2024BVD16988	Carnivore	Red fox	*Vulpes vulpes*	Vorpommern-Rügen	98	negative	-	-	-	negative	negative	negative
2024BVD16989	Carnivore	Red fox	*Vulpes vulpes*	Vorpommern-Rügen	52	negative	-	-	-	negative	negative	negative
2024BVD16990	Carnivore	Red fox	*Vulpes vulpes*	Vorpommern-Rügen	68	negative	-	-	-	negative	negative	negative
2024BVD16991	Carnivore	Raccoon dog	*Nyctereutes procyonoides*	Vorpommern-Rügen	106	negative	-	-	-	negative	negative	negative
2024BVD16992	Carnivore	Raccoon dog	*Nyctereutes procyonoides*	Vorpommern-Rügen	110	negative	-	-	-	negative	negative	negative
2024BVD16993	Carnivore	Red fox	*Vulpes vulpes*	Vorpommern-Rügen	65	negative	-	-	-	negative	negative	negative
2024BVD16994	Carnivore	Raccoon dog	*Nyctereutes procyonoides*	Vorpommern-Rügen	not taken	not taken	not taken	not taken	-	negative	negative	negative
2024BVD16995	Carnivore	Raccoon dog	*Nyctereutes procyonoides*	Vorpommern-Rügen	96	negative	-	-	-	negative	negative	negative

Sample ID	Group	Species	Scientific species name	Sample origin rural district	Result IAV-NP antibody ELISA in percent*	Assessment IAV-NP antibody ELISA	Result IAV-H5 antibody ELISA in percent†	Assessment IAV-H5 antibody ELISA	Titer serum neutralization assay	IAV RT-qPCR nasal swab	IAV RT-qPCR lung	IAV RT-qPCR brain
2024BVD16996	Carnivore	Raccoon dog	*Nyctereutes procyonoides*	Vorpommern-Rügen	not taken	not taken	not taken	not taken	-	negative	negative	negative
2024BVD16997	Carnivore	Red fox	*Vulpes vulpes*	Vorpommern-Rügen	104	negative	-	-	-	negative	negative	negative
2024BVD16998	Carnivore	Raccoon	*Procyon lotor*	Vorpommern-Rügen	109	negative	-	-	-	negative	negative	negative
2024BVD16999	Carnivore	Raccoon	*Procyon lotor*	Vorpommern-Rügen	105	negative	-	-	-	negative	negative	negative
2024BVD17000	Carnivore	Raccoon	*Procyon lotor*	Vorpommern-Rügen	105	negative	-	-	-	negative	negative	not taken
2024BVD17001	Carnivore	Red fox	*Vulpes vulpes*	Vorpommern-Rügen	101	negative	-	-	-	negative	negative	negative
2024BVD17002	Carnivore	Raccoon	*Procyon lotor*	Vorpommern-Rügen	105	negative	-	-	-	negative	negative	negative
2024BVD17003	Carnivore	Raccoon	*Procyon lotor*	Vorpommern-Rügen	105	negative	-	-	-	negative	negative	negative
2024BVD17004	Carnivore	Red fox	*Vulpes vulpes*	Vorpommern-Rügen	106	negative	-	-	-	negative	negative	negative
2024BVD17005	Carnivore	Red fox	*Vulpes vulpes*	Vorpommern-Rügen	94	negative	-	-	-	negative	negative	negative
2024BVD17006	Carnivore	Red fox	*Vulpes vulpes*	Vorpommern-Rügen	109	negative	-	-	-	negative	negative	negative
2024BVD17007	Carnivore	Raccoon dog	*Nyctereutes procyonoides*	Vorpommern-Rügen	103	negative	-	-	-	negative	negative	negative
2024BVD17008	Carnivore	Raccoon dog	*Nyctereutes procyonoides*	Vorpommern-Rügen	67	negative	-	-	-	negative	negative	negative
2024BVD17009	Carnivore	Red fox	*Vulpes vulpes*	Vorpommern-Rügen	97	negative	-	-	-	negative	negative	negative
2024BVD17010	Carnivore	Raccoon	*Procyon lotor*	Vorpommern-Rügen	109	negative	-	-	-	negative	negative	negative
2024BVD17011	Carnivore	Raccoon dog	*Nyctereutes procyonoides*	Vorpommern-Rügen	92	negative	-	-	-	negative	negative	negative
2024BVD17012	Carnivore	Red fox	*Vulpes vulpes*	Vorpommern-Rügen	101	negative	-	-	-	negative	negative	negative
2024BVD17013	Carnivore	Raccoon dog	*Nyctereutes procyonoides*	Vorpommern-Rügen	95	negative	-	-	-	negative	negative	negative
2024BVD17014	Carnivore	Raccoon	*Procyon lotor*	Vorpommern-Rügen	94	negative	-	-	-	negative	negative	negative
2024BVD17015	Carnivore	Red fox	*Vulpes vulpes*	Vorpommern-Rügen	99	negative	-	-	-	negative	negative	negative
2024BVD17294	Carnivore	Red fox	*Vulpes vulpes*	Vorpommern-Rügen	64	negative	-	-	-	negative	negative	negative
2024BVD17295	Carnivore	Red fox	*Vulpes vulpes*	Vorpommern-Rügen	99	negative	-	-	-	negative	negative	negative
2024BVD17296	Carnivore	Red fox	*Vulpes vulpes*	Vorpommern-Rügen	46	undetermined	not available	not available	-	negative	not taken	negative
2024BVD17297	Carnivore	Raccoon	*Procyon lotor*	Vorpommern-Rügen	98	negative	-	-	-	negative	negative	negative
2024BVD17298	Carnivore	Red fox	*Vulpes vulpes*	Vorpommern-Rügen	66	negative	-	-	-	negative	negative	negative
2024BVD17299	Carnivore	Raccoon dog	*Nyctereutes procyonoides*	Vorpommern-Rügen	90	negative	-	-	-	negative	negative	negative
2024BVD17300	Carnivore	Raccoon dog	*Nyctereutes procyonoides*	Vorpommern-Rügen	101	negative	-	-	-	negative	negative	negative
2024BVD17301	Carnivore	Red fox	*Vulpes vulpes*	Vorpommern-Rügen	not taken	not taken	not taken	not taken	-	negative	negative	negative
2024BVD17302	Carnivore	Red fox	*Vulpes vulpes*	Vorpommern-Rügen	not taken	not taken	not taken	not taken	-	negative	negative	negative
2024BVD17303	Carnivore	Red fox	*Vulpes vulpes*	Vorpommern-Rügen	74	negative	-	-	-	negative	negative	negative
2024BVD17304	Carnivore	Raccoon dog	*Nyctereutes procyonoides*	Vorpommern-Rügen	81	negative	-	-	-	negative	not taken	negative
2024BVD17305	Carnivore	Red fox	*Vulpes vulpes*	Vorpommern-Rügen	38	positive	8	positive	-	negative	negative	negative
2024BVD17306	Carnivore	Red fox	*Vulpes vulpes*	Vorpommern-Rügen	98	negative	-	-	-	negative	negative	negative
2024BVD17307	Carnivore	Raccoon	*Procyon lotor*	Vorpommern-Rügen	98	negative	-	-	-	negative	negative	negative
2024BVD17308	Carnivore	Red fox	*Vulpes vulpes*	Vorpommern-Rügen	99	negative	-	-	-	negative	negative	negative

Sample ID	Group	Species	Scientific species name	Sample origin rural district	Result IAV-NP antibody ELISA in percent*	Assessment IAV-NP antibody ELISA	Result IAV-H5 antibody ELISA in percent†	Assessment IAV-H5 antibody ELISA	Titer serum neutralization assay	IAV RT-qPCR nasal swab	IAV RT-qPCR lung	IAV RT-qPCR brain
2024BVD17309	Carnivore	Raccoon dog	*Nyctereutes procyonoides*	Vorpommern-Rügen	98	negative	-	-	-	negative	negative	not taken
2024BVD17310	Carnivore	Raccoon dog	*Nyctereutes procyonoides*	Vorpommern-Rügen	not taken	not taken	not taken	not taken	-	negative	not taken	not taken
2024BVD17311	Carnivore	Red fox	*Vulpes vulpes*	Vorpommern-Rügen	96	negative	-	-	-	negative	negative	negative
2024BVD17312	Carnivore	Red fox	*Vulpes vulpes*	Vorpommern-Rügen	103	negative	-	-	-	negative	negative	negative
2024BVD17313	Carnivore	Red fox	*Vulpes vulpes*	Vorpommern-Rügen	84	negative	-	-	-	negative	negative	negative
2024BVD17314	Carnivore	Red fox	*Vulpes vulpes*	Vorpommern-Rügen	88	negative	-	-	-	negative	negative	negative
2024BVD17315	Carnivore	Red fox	*Vulpes vulpes*	Vorpommern-Rügen	95	negative	-	-	-	negative	negative	negative
2024BVD17316	Carnivore	Raccoon dog	*Nyctereutes procyonoides*	Vorpommern-Rügen	70	negative	-	-	-	negative	negative	negative
2024BVD17317	Carnivore	Raccoon dog	*Nyctereutes procyonoides*	Vorpommern-Rügen	88	negative	-	-	-	negative	negative	negative
2024BVD17318	Carnivore	European badger	*Meles meles*	Vorpommern-Rügen	55	negative	-	-	-	negative	negative	not taken
2024BVD17319	Carnivore	Red fox	*Vulpes vulpes*	Vorpommern-Rügen	35	positive	30	positive	-	negative	negative	negative
2024BVD17320	Carnivore	Red fox	*Vulpes vulpes*	Vorpommern-Rügen	not taken	not taken	not taken	not taken	-	negative	negative	negative
2024BVD17321	Carnivore	Raccoon	*Procyon lotor*	Vorpommern-Rügen	26	positive	7	positive	-	negative	negative	negative
2024BVD17322	Carnivore	Red fox	*Vulpes vulpes*	Vorpommern-Rügen	63	negative	-	-	-	negative	negative	negative
2024BVD17323	Carnivore	Raccoon dog	*Nyctereutes procyonoides*	Vorpommern-Rügen	50	undetermined	23	positive	-	negative	negative	not taken
2024BVD17324	Carnivore	Red fox	*Vulpes vulpes*	Vorpommern-Rügen	71	negative	-	-	-	negative	negative	negative
2024BVD17325	Carnivore	Raccoon dog	*Nyctereutes procyonoides*	Vorpommern-Rügen	not taken	not taken	not taken	not taken	-	negative	negative	negative
2024BVD17326	Carnivore	Raccoon dog	*Nyctereutes procyonoides*	Vorpommern-Rügen	not taken	not taken	not taken	not taken	-	negative	negative	negative
2024BVD17327	Carnivore	Red fox	*Vulpes vulpes*	Vorpommern-Rügen	20	positive	30	positive	-	negative	negative	negative
2024BVD17328	Carnivore	Red fox	*Vulpes vulpes*	Vorpommern-Rügen	not taken	not taken	not taken	not taken	-	negative	negative	negative
2024BVD17329	Carnivore	Red fox	*Vulpes vulpes*	Vorpommern-Rügen	69	negative	-	-	-	negative	negative	negative
2024BVD17330	Carnivore	Raccoon	*Procyon lotor*	Vorpommern-Rügen	16	positive	30	positive	-	negative	negative	negative
2024BVD17331	Carnivore	Raccoon dog	*Nyctereutes procyonoides*	Vorpommern-Rügen	not taken	not taken	not taken	not taken	-	negative	negative	negative
2024BVD17332	Carnivore	Raccoon dog	*Nyctereutes procyonoides*	Vorpommern-Rügen	not taken	not taken	not taken	not taken	-	negative	negative	negative
2024BVD17333	Carnivore	Raccoon dog	*Nyctereutes procyonoides*	Vorpommern-Rügen	106	negative	-	-	-	negative	negative	negative
2024BVD17334	Carnivore	Raccoon dog	*Nyctereutes procyonoides*	Vorpommern-Rügen	79	negative	-	-	-	negative	negative	negative
2024BVD17807	Carnivore	Red fox	*Vulpes vulpes*	Vorpommern-Rügen	73	negative	-	-	-	negative	negative	negative
2024BVD17808	Carnivore	Red fox	*Vulpes vulpes*	Vorpommern-Rügen	28	positive	5	positive	-	negative	negative	negative
2024BVD17809	Carnivore	Raccoon dog	*Nyctereutes procyonoides*	Vorpommern-Rügen	62	negative	-	-	-	negative	negative	negative
2024BVD17810	Carnivore	Raccoon dog	*Nyctereutes procyonoides*	Vorpommern-Rügen	65	negative	-	-	-	negative	negative	negative
2024BVD17811	Carnivore	Red fox	*Vulpes vulpes*	Vorpommern-Rügen	66	negative	-	-	-	negative	negative	negative

Sample ID	Group	Species	Scientific species name	Sample origin rural district	Result IAV-NP antibody ELISA in percent*	Assessment IAV-NP antibody ELISA	Result IAV-H5 antibody ELISA in percent†	Assessment IAV-H5 antibody ELISA	Titer serum neutralization assay	IAV RT-qPCR nasal swab	IAV RT-qPCR lung	IAV RT-qPCR brain
2024BVD17812	Carnivore	Red fox	*Vulpes vulpes*	Vorpommern-Rügen	42	positive	70	negative	-	negative	negative	negative
2024BVD17813	Carnivore	Red fox	*Vulpes vulpes*	Vorpommern-Rügen	89	negative	-	-	-	negative	negative	negative
2024BVD17814	Carnivore	Red fox	*Vulpes vulpes*	Vorpommern-Rügen	not taken	not taken	not taken	not taken	-	negative	negative	negative
2024BVD17815	Carnivore	Raccoon	*Procyon lotor*	Vorpommern-Rügen	50	undetermined	43	positive	-	negative	negative	negative
2024BVD17816	Carnivore	Red fox	*Vulpes vulpes*	Vorpommern-Rügen	46	undetermined	50 (49.7)	positive	-	negative	negative	negative
2024BVD17817	Carnivore	Raccoon	*Procyon lotor*	Vorpommern-Rügen	70	negative	-	-	-	negative	negative	negative
2024BVD17818	Carnivore	Red fox	*Vulpes vulpes*	Vorpommern-Rügen	60	negative	-	-	-	negative	negative	negative
2024BVD17819	Carnivore	Red fox	*Vulpes vulpes*	Vorpommern-Rügen	69	negative	-	-	-	negative	negative	negative
2024BVD17820	Carnivore	Raccoon	*Procyon lotor*	Vorpommern-Rügen	73	negative	-	-	-	negative	negative	negative
2024BVD17821	Carnivore	Red fox	*Vulpes vulpes*	Vorpommern-Rügen	16	positive	16	positive	-	negative	negative	negative
2024BVD17822	Carnivore	Fox	*Vulpes sp.*	Vorpommern-Rügen	17	positive	18	positive	-	negative	negative	negative
2024BVD17823	Carnivore	Fox	*Vulpes sp.*	Vorpommern-Rügen	12	positive	8	positive	-	negative	negative	negative
2024BVD17824	Carnivore	Fox	*Vulpes sp.*	Vorpommern-Rügen	66	negative	-	-	-	negative	negative	negative
2025BVD00300	Carnivore	Fox	*Vulpes sp.*	Vorpommern-Rügen	45	undetermined	25	positive	-	negative	negative	negative
2025BVD00301	Carnivore	Raccoon dog	*Nyctereutes procyonoides*	Vorpommern-Rügen	12	positive	17	positive	-	negative	negative	negative
2025BVD00302	Carnivore	Fox	*Vulpes sp.*	Vorpommern-Rügen	23	positive	61	negative	<1:20	negative	negative	negative
2025BVD00303	Carnivore	Fox	*Vulpes sp.*	Vorpommern-Rügen	57	negative	-	-	-	negative	negative	negative
2025BVD00304	Carnivore	Raccoon	*Procyon lotor*	Vorpommern-Rügen	71	negative	-	-	-	negative	negative	negative
2025BVD00305	Carnivore	Raccoon dog	*Nyctereutes procyonoides*	Vorpommern-Rügen	72	negative	-	-	-	negative	negative	negative
2025BVD00306	Carnivore	Fox	*Vulpes sp.*	Vorpommern-Rügen	57	negative	-	-	-	negative	negative	negative
2025BVD00307	Carnivore	Fox	*Vulpes sp.*	Vorpommern-Rügen	63	negative	-	-	-	negative	negative	not taken
2025BVD00308	Carnivore	Fox	*Vulpes sp.*	Vorpommern-Rügen	32	positive	52	undetermined	-	negative	negative	negative
2025BVD00309	Carnivore	Raccoon	*Procyon lotor*	Vorpommern-Rügen	10	positive	7	positive	-	negative	negative	negative
2025BVD00310	Carnivore	Fox	*Vulpes sp.*	Vorpommern-Rügen	13	positive	5	positive	-	negative	negative	not taken
2025BVD00311	Carnivore	Raccoon dog	*Nyctereutes procyonoides*	Vorpommern-Rügen	12	positive	8	positive	-	negative	negative	not taken
2025BVD00312	Carnivore	Fox	*Vulpes sp.*	Vorpommern-Rügen	75	negative	-	-	-	negative	negative	negative
2025BVD00313	Carnivore	Raccoon dog	*Nyctereutes procyonoides*	Vorpommern-Rügen	81	negative	-	-	-	negative	negative	not taken
2025BVD00314	Carnivore	Fox	*Vulpes sp.*	Vorpommern-Rügen	76	negative	-	-	-	negative	negative	negative
2025BVD00315	Carnivore	Raccoon dog	*Nyctereutes procyonoides*	Vorpommern-Rügen	70	negative	-	-	-	negative	negative	negative
2025BVD00316	Carnivore	Raccoon	*Procyon lotor*	Vorpommern-Rügen	77	negative	-	-	-	negative	negative	not taken
2025BVD00317	Carnivore	Raccoon	*Procyon lotor*	Vorpommern-Rügen	60	negative	-	-	-	negative	negative	negative
2025BVD00318	Carnivore	Raccoon dog	*Nyctereutes procyonoides*	Vorpommern-Rügen	44	positive	75	negative	-	negative	negative	negative
2025BVD00319	Carnivore	Fox	*Vulpes sp.*	Vorpommern-Rügen	85	negative	-	-	-	negative	negative	negative
2025BVD00320	Carnivore	Fox	*Vulpes sp.*	Vorpommern-Rügen	74	negative	-	-	-	negative	negative	negative
2025BVD00321	Carnivore	Raccoon dog	*Nyctereutes procyonoides*	Vorpommern-Rügen	20	positive	7	positive	-	negative	negative	negative
2025BVD00322	Carnivore	Fox	*Vulpes sp.*	Vorpommern-Rügen	70	negative	-	-	-	negative	negative	negative

Sample ID	Group	Species	Scientific species name	Sample origin rural district	Result IAV-NP antibody ELISA in percent*	Assessment IAV-NP antibody ELISA	Result IAV-H5 antibody ELISA in percent†	Assessment IAV-H5 antibody ELISA	Titer serum neutralization assay	IAV RT-qPCR nasal swab	IAV RT-qPCR lung	IAV RT-qPCR brain
2025BVD00323	Carnivore	Raccoon dog	*Nyctereutes procyonoides*	Vorpommern-Rügen	20	positive	6	positive	1:60	negative	negative	negative
2025BVD00324	Carnivore	Fox	*Vulpes sp.*	Vorpommern-Rügen	53	negative	-	-	-	negative	negative	negative
2025BVD00325	Carnivore	Fox	*Vulpes sp.*	Vorpommern-Rügen	63	negative	-	-	-	negative	negative	not taken
2025BVD00326	Carnivore	Fox	*Vulpes sp.*	Vorpommern-Rügen	not taken	not taken	not taken	not taken	-	negative	negative	negative
2025BVD00327	Carnivore	Fox	*Vulpes sp.*	Vorpommern-Rügen	24	positive	28	positive	-	negative	negative	negative
2025BVD00328	Carnivore	Fox	*Vulpes sp.*	Vorpommern-Rügen	63	negative	-	-	-	negative	negative	negative
2025BVD00329	Carnivore	Fox	*Vulpes sp.*	Vorpommern-Rügen	58	negative	-	-	-	negative	negative	negative
2025BVD00330	Carnivore	Fox	*Vulpes sp.*	Vorpommern-Rügen	67	negative	-	-	-	negative	negative	negative
2025BVD00847	Carnivore	Raccoon dog	*Nyctereutes procyonoides*	Vorpommern-Rügen	73	negative	-	-	-	negative	negative	negative
2025BVD00848	Carnivore	Fox	*Vulpes sp.*	Vorpommern-Rügen	96	negative	-	-	-	negative	negative	negative
2025BVD00849	Carnivore	Fox	*Vulpes sp.*	Vorpommern-Rügen	not taken	not taken	not taken	not taken	-	negative	negative	negative
2025BVD00850	Carnivore	Fox	*Vulpes sp.*	Vorpommern-Rügen	43	positive	75	negative	-	negative	negative	negative
2025BVD00851	Carnivore	Fox	*Vulpes sp.*	Vorpommern-Rügen	52	negative	-	-	-	negative	negative	negative
2025BVD00852	Carnivore	Raccoon	*Procyon lotor*	Vorpommern-Rügen	33	positive	70	negative	-	negative	negative	negative
2025BVD00853	Carnivore	Raccoon dog	*Nyctereutes procyonoides*	Vorpommern-Rügen	83	negative	-	-	-	negative	negative	negative
2025BVD00854	Carnivore	Fox	*Vulpes sp.*	Vorpommern-Rügen	56	negative	-	-	-	negative	negative	negative
2025BVD00855	Carnivore	Fox	*Vulpes sp.*	Vorpommern-Rügen	59	negative	-	-	-	negative	negative	negative
2025BVD00856	Carnivore	Fox	*Vulpes sp.*	Vorpommern-Rügen	53	negative	-	-	-	negative	negative	negative
2025BVD00857	Carnivore	Raccoon dog	*Nyctereutes procyonoides*	Vorpommern-Rügen	38	positive	68	negative	-	negative	negative	negative
2025BVD00858	Carnivore	Raccoon	*Procyon lotor*	Vorpommern-Rügen	63	negative	-	-	-	negative	negative	negative
2025BVD00859	Carnivore	Fox	*Vulpes sp.*	Vorpommern-Rügen	not taken	not taken	not taken	not taken	-	negative	negative	negative
2025BVD00860	Carnivore	Fox	*Vulpes sp.*	Vorpommern-Rügen	29	positive	6	positive	-	negative	negative	negative
2025BVD00861	Carnivore	Fox	*Vulpes sp.*	Vorpommern-Rügen	72	negative	-	-	-	negative	negative	negative
2025BVD00862	Carnivore	Fox	*Vulpes sp.*	Vorpommern-Rügen	34	positive	8	positive	-	negative	negative	negative
2025BVD00863	Carnivore	Fox	*Vulpes sp.*	Vorpommern-Rügen	52	negative	-	-	-	negative	negative	negative
2025BVD00864	Carnivore	Fox	*Vulpes sp.*	Vorpommern-Rügen	91	negative	-	-	-	negative	negative	negative
2025BVD00865	Carnivore	Fox	*Vulpes sp.*	Vorpommern-Rügen	65	negative	-	-	-	negative	negative	negative
2025BVD00866	Carnivore	Raccoon dog	*Nyctereutes procyonoides*	Vorpommern-Rügen	57	negative	-	-	-	negative	negative	negative
2025BVD00867	Carnivore	Raccoon dog	*Nyctereutes procyonoides*	Vorpommern-Rügen	35	positive	23	positive	-	negative	negative	negative
2025BVD00868	Carnivore	Raccoon dog	*Nyctereutes procyonoides*	Vorpommern-Rügen	76	negative	-	-	-	negative	not taken	negative
2025BVD00869	Carnivore	Fox	*Vulpes sp.*	Vorpommern-Rügen	8	positive	6	positive	-	negative	negative	negative
2025BVD00870	Carnivore	Fox	*Vulpes sp.*	Vorpommern-Rügen	66	negative	-	-	-	negative	negative	negative
2025BVD00871	Carnivore	Raccoon	*Procyon lotor*	Vorpommern-Rügen	63	negative	-	-	-	negative	negative	negative
2025BVD00872	Carnivore	Fox	*Vulpes sp.*	Vorpommern-Rügen	65	negative	-	-	-	negative	negative	negative
2025BVD00873	Carnivore	Fox	*Vulpes sp.*	Vorpommern-Rügen	61	negative	-	-	-	negative	negative	negative
2025BVD00874	Carnivore	Fox	*Vulpes sp.*	Vorpommern-Rügen	24	positive	24	positive	-	negative	negative	negative
2025BVD00875	Carnivore	Fox	*Vulpes sp.*	Vorpommern-Rügen	75	negative	-	-	-	negative	negative	negative

Sample ID	Group	Species	Scientific species name	Sample origin rural district	Result IAV-NP antibody ELISA in percent*	Assessment IAV-NP antibody ELISA	Result IAV-H5 antibody ELISA in percent†	Assessment IAV-H5 antibody ELISA	Titer serum neutralization assay	IAV RT-qPCR nasal swab	IAV RT-qPCR lung	IAV RT-qPCR brain
2025BVD00876	Carnivore	Fox	*Vulpes sp.*	Vorpommern-Rügen	38	positive	17	positive	-	negative	negative	negative
2025BVD00877	Carnivore	Fox	*Vulpes sp.*	Vorpommern-Rügen	66	negative	-	-	-	negative	negative	negative
2025BVD00878	Carnivore	Raccoon dog	*Nyctereutes procyonoides*	Vorpommern-Rügen	70	negative	-	-	-	negative	negative	negative

*Cut-off values for IAV-NP antibody ELISA in percent are considered positive ≤ 45%, undetermined 45% < result < 50% and negative ≥ 50% according to the manufacturer's instructions
†Cut-off values for IAV-H5 antibody ELISA in percent are considered positive ≤ 50%, undetermined 50% < result < 60% and negative ≥ 60% according to the manufacturer's instructions

Appendix Table 2. Environmental variables tested either as proportion (variables ending with "prop") in a 2.5 km buffer zone or as a distance in 100 m steps (variables ending with "dist100") to the point geographic location where the animal carcass had been found.

Variable (abbreviation used in modelling)	Short description	Detailed description, including codes of the „Biotoptypen- und Nutzungstypenkartierung"
Forest (Forest_prop, Forest_dist100)	forest (< 4 ha)	B10 Forest (> 4 ha) B11 Deciduous forest B12 Mixed deciduous forest (< 10 % conifers) B13 Mixed deciduous forest (ratio of deciduous to coniferous trees 90/10 - 70/30) B14 Mixed forest (ratio of deciduous to coniferous trees 50/50) B15 Mixed coniferous forest (ratio of coniferous to deciduous trees 90/10 - 70/30) B16 Coniferous forest B17 Forest edge B18 Clear-cut B19 Glade/clearing
Shrubland (Shrubland_prop, Shrubland_dist100)	group of trees, hedge, bushes	B20 Group of trees, hedge, bushes B21 Field copse (0.5 - 4 ha) B22 Group of trees (< 0.5 ha) B23 Row of trees B24 Avenue B25 Dominant single tree B26 Hedge B27 Bushes, group of shrubs
Farmland (Farmland_prop, Farmland_dist100)	arable land, commercial horticulture	L20 Arable land, commercial horticulture L21 Arable land L22 Commercial horticulture L23 Tree nursery L24 Fruit growing
Grassland (Grassland_prop, Grassland_dist100)	Grassland	L11 Wet grassland L12 Fresh grassland bntk_f.doc Page 3 27.02.2013 L13 Seasonally wet grassland L14 Dry grassland L15 Salt grassland
Open areas (Openareas_prop, Openareas_dist100)	open space	S40 Open space S41 Park S42 Wildlife enclosure, zoo S43 Leisure park S44 Sports facility S45 Golf course S46 Campsite S47 Allotment gardens, holiday homes S48 Village green S49 Cemetery
Transportation (Trafficarea_prop, Trafficarea_dist100)	traffic area	S50 Traffic area S51 Path S52 Unpaved farm track S53 Paved farm track S54 Road S55 Motorway S56 Railway/Railway system S57 Airfield S58 Port facility S59 Parking lot
Watercourses (Watercourses_prop, Watercourses_dist100)	Rivers	W10 Flowing water W11 Source area W12 Stream < 3m W13 Ditch < 3m W14 River > 3m W15 Canal > 3m
Excavation (Excavation_prop, Excavation_dist100)	excavation and embankment	R00 Excavation and backfill R10 Raw material extraction R11 Quarry/chalk quarry R12 Sand/gravel pit R13 Clay pit R14 Undifferentiated site excavation R20 Backfill R21 Landfill R22 Uncontrolled landfill R23 Scour field R24 Agricultural storage area (dung heap, pile, silo)
Heathland (Heathland_prop, Heathland_dist100)	heath	T10 Heide T11 Zwergstrauchheide T12 Ginsterheide T13 Krähenbeerheide
Dryland (Dryland_prop, Dryland_dist100)	dry grassland	T20 dry grassland T21 silicate dry grassland T22 calcareous dry grassland
	rocky outcrop	T30 Rocky outcrop T31 Inset outcrop rock T32 Stone pile and wall T33 Single boulder
Infrastructure (Infrastruct_prop, Infrastruct_dist100)	residential area	S10 Residential area S11 Closed development S12 Individual development S13 New development area, undifferentiated
	mixed area	S20 Mixed-use area S21 Urban mixed-use area S22 Rural mixed-use area S23 Single farmstead
	production facility	S30 Production facility S31 Commercial and industrial area S32 Animal production facility S33 Military facility
	hydraulic structure	S60 Hydraulic structure S61 Dike, dam S62 Groyne S63 Stone wall S64 Pumping station
	supply and disposal facilities	S70 Supply and disposal facilities S71 District heating pipeline S72 Sewage treatment plant S73 Slurry tank
Other waterbodies (Otherwater_prop, Otherwater_dist100)	small standing water body < 1 ha	W20 Small standing water body < 1 ha W21 Temporary small water body, pond or pool W22 Permanent small water body
	standing water > 1 ha	W30 Standing water > 1 ha W31 Shallow lake < 5m W32 Lake
	moor and swamp	W40 Moor and swamp W41 Fens W42 Raised and transitional moor W43 Swamp
	coastal biotopes	W70 Coastal biotopes W71 Sand hook W72 Beach W73 Beach lake W74 Beach wall W75 White dune W76 Cliff W77 Cliff edge dune
Bodden coast (Baycoast_prop, Baycoast_dist100)	bodden (bay)	W60 Bodden (bay) W61 Open water [Bodden] W62 Marine boulder and stone bottom [Bodden] W63 Sandbank [Bodden]
Urban (Urban_prop, Urban_dist)	DLM data	mixed use, cemetery, industry and commerce, sports / leisure / recreation, residential area, square, road traffic, traffic structures, open-cast mine / pit / quarry, rail traffic, air traffic, spoil heap
Baltic sea (Balticsea_prop, Balticsea_dist)	this study	all remaining areas surrounding the island

Appendix Table 3. Results of the univariable logistic regression analysis using data on landcover and individual animals (for land cover data on buffer proportions [prop] or distance in 100 m [dist100] were used; data on age was stratified for adult and juvenile foxes). Results displayed were restricted to models with P values < 0.2.

Model (AIC[a])	Variable	Estimate	Standard Error	z-value	*P*-value	Odds Ratio	2.5% CI[b]	97.5% CI[b]	Category P-value (< 0.05)[c]
1 (269.12)	(Intercept)	-1.58	0.20	-7.81	5.76E-15	0.21	0.14	0.30	***
	Baycoast_prop	1.22	0.88	1.39	0.166	3.37	0.57	19.10	-
2 (268.55)	(Intercept)	-1.16	0.21	-5.42	5.91E-08	0.31	0.20	0.47	***
	Otherwater_prop	-1.57	1.06	-1.48	0.139	0.21	0.02	1.46	-
3 (265.17)	(Intercept)	-0.81	0.29	-2.80	0.0052	0.45	0.25	0.78	**
	Shrubland_prop	-2.89	1.28	-2.25	0.0245	0.06	0.00	0.60	*
4 (267.96)	(Intercept)	-1.17	0.20	-5.85	4.93E-09	0.31	0.21	0.46	***
	Forest_prop	-0.28	0.17	-1.61	0.107	0.76	0.52	1.03	-
5 (267.97)	(Intercept)	-1.61	0.20	-8.07	6.98E-16	1.99E-01	0.13	0.29	***
	Openareas_prop	19.54	10.97	1.78	0.075	3.07E+08	0.07	5.16E+17	.
6 (268.4)	(Intercept)	-1.27	0.17	-7.29	3.20E-13	0.28	0.20	0.39	***
	Excavation_prop	-2.89	2.03	-1.43	0.154	0.06	0.00	1.74	-
7 (268.49)	(Intercept)	-1.31	0.16	-7.99	1.37E-15	0.27	1.95E-01	0.37	***
	Dryland_prop	-4.45	3.17	-1.40	0.161	0.01	7.29E-06	2.55	-
8 (267.66)	(Intercept)	-1.10	0.22	-5.01	5.36E-07	3.32E-01	2.13E-01	0.51	***
	Trafficarea_prop	-10.69	6.01	-1.78	0.0755	2.29E-05	1.15E-10	2.18	.
9 (265.7)	(Intercept)	-0.87	0.28	-3.05	0.00231	0.42	0.24	0.74	**
	Urban_prop	-1.16	0.56	-2.06	0.03945	0.31	0.09	0.86	*
10 (264.86)	(Intercept)	-1.66	0.19	-8.72	<2e-16	1.90E-01	0.13	0.27	***
	Watercourses_prop	31.65	12.57	2.52	0.0118	5.54E+13	917.99	6.49E+24	*
11 (269.26)	(Intercept)	-1.52	0.18	-8.48	<2e-16	0.22	0.15	0.31	***
	Greenland_dist100	0.06	0.05	1.31	0.19	1.06	0.97	1.18	-
12 (269.27)	(Intercept)	-1.51	0.17	-8.66	<2e-16	0.22	0.16	0.31	***
	Srubland_dist100	0.04	0.03	1.30	0.195	1.04	0.98	1.11	-
13 (269.24)	(Intercept)	-1.62	0.23	-7.17	7.40E-13	0.20	0.13	0.31	***
	Urban_dist100	0.06	0.04	1.36	0.175	1.06	0.97	1.15	-
14 (263.91)	(Intercept)	-0.99	0.21	-4.65	3.34E-06	0.37	0.24	0.56	***
	Baycoast_dist100	-0.02	0.01	-2.49	0.0128	0.98	0.96	1.00	*
15 (262.91)	(Intercept)	-0.93	0.22	-4.23	2.37E-05	0.39	0.25	0.60	***
	Balticsea_dist100	-0.03	0.01	-2.60	0.00935	0.97	0.94	0.99	**
16 (264.26)	(Intercept)	-0.84	0.26	-3.23	0.00126	0.43	0.26	0.71	**
	Watercourses_dist100	-0.05	0.02	-2.43	0.01527	0.96	0.92	0.99	*
17 (265.37)	(Intercept)	-1.18	0.17	-6.77	1.28E-11	0.31	0.22	0.43	***
	Age-Adult (Ref.)	-	-	-	-	-	-	-	-
	Age-Juvenil	-0.85	0.38	-2.24	0.0253	0.43	0.19	0.87	*

[a] Akaike Information Index, [b]2.5% and 97.5% Confidence interval of Odds ratio, [c] *=P-value < 0.05, **= P-value < 0.01, ***= P-value < 0.001

Appendix Table 4. Final multivariable logistic regression model using landcover variables (buffer proportions (prop), distance in 100 m (dist100) or age (adult vs juvenile).

Model (AIC[a])	Variable	Esti-mate	Standard Error	z-value	*P*-value	Odds Ratio	2.5% CI[b]	97.5% CI[b]	Category P-value (< 0.05)[c]
18 (248.75)	(Intercept)	0.37	0.45	0.81	0.42	1.44	0.60	3.60	-
	Shrubland_prop	-4.11	1.54	-2.66	0.01	0.02	0.00	0.29	**
	Shrubland_dist100	0.11	0.04	2.67	0.01	1.11	1.02	1.20	**
	Age-Adult (Ref.)	-	-	-	-	-	-	-	-
	Age-Juvenil	-0.55	0.40	-1.38	0.17	0.58	0.25	1.23	
	Watercourses_dist100	-0.09	0.02	-3.89	0.00	0.92	0.88	0.96	***

[a] Akaike Information Index, [b]2.5% and 97.5% Confidence interval of Odds ratio, [c] **= P-value < 0.01, ***= P-value < 0.001
